# Unraveling Reactivity Pathways: Dihydrogen Activation and Hydrogenation of Multiple Bonds by Pyramidalized Boron‐Based Frustrated Lewis Pairs

**DOI:** 10.1002/open.202300179

**Published:** 2023-12-20

**Authors:** Himangshu Mondal, Pratim Kumar Chattaraj

**Affiliations:** ^1^ Department of Chemistry Indian Institute of Technology Kharagpur 721302 India; ^2^ Department of Chemistry Birla Institute of Technology Mesra Ranchi 835215 Jharkhand India

**Keywords:** Activation strain model, Density functional theory, Energy decomposition analysis, Frustrated Lewis pairs, Main group chemistry

## Abstract

The activation of H_2_ by pyramidalized boron‐based frustrated Lewis Pairs (FLPs) (**B/E‐FLP** systems where “**E**” refers to N, P, As, Sb, and Bi) have been explored using density functional theory (DFT) based computational study. The activation pathway for the entire process is accurately characterized through the utilization of the activation strain model (ASM) of reactivity, shedding light on the underlying physical factors governing the process. The study also explores the hydrogenation process of multiple bonds with the help of B/N‐FLP. The research findings demonstrate that the liberation of activated dihydrogen occurs in a synchronized, albeit noticeably asynchronous, fashion. The transformation is extensively elucidated using the activation strain model and the energy decomposition analysis. This approach suggests a co‐operative double hydrogen‐transfer mechanism, where the B−H hydride triggers a nucleophilic attack on the carbon atom of the multiple bonds, succeeded by the migration of the protic N−H.

## Graphical abstract

A DFT‐based study explores the H_2_ activation by pyramidalized boron‐based B/E‐FLP (E=N, P, As, Sb and Bi, etc.) systems. The study also highlights the hydrogenation process of multiple bonds with the help of B/N‐FLP.

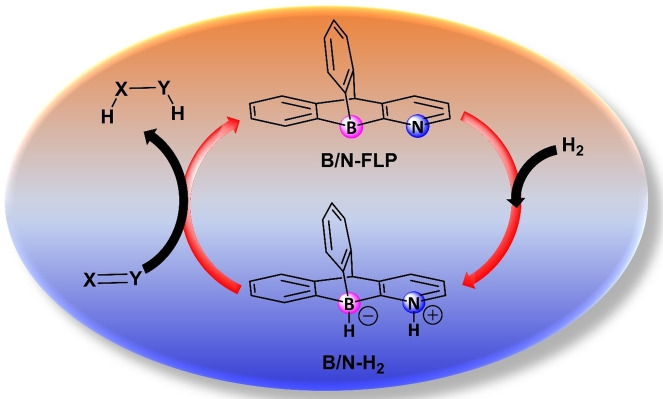

## Introduction

Transition metal chemistry has long been the domain of dihydrogen activation,[^1^, ^2^] even nature itself harnesses metal‐centered reactions to split the dihydrogen molecule within hydrogenase enzymes.^[3]^ However, a fascinating breakthrough has recently emerged as a shift from metal‐based systems to metal‐free activation approaches. Ever since Stephan et al. made a significant breakthrough in 2006 by discovering the activation of dihydrogen using sterically hindered pairs of Lewis acid and Lewis base,^[4]^ the field of frustrated Lewis pairs (FLPs) has made remarkable progress. In particular, phosphane/borane Lewis pairs have shown rapid and efficient dihydrogen activation, yielding intriguing phosphonium cation/hydridoborate anion pairs. In the past ten years, extensive research has been conducted on different aspects related to FLPs. Those include the activation of small,[^5^, ^6^, ^7^, ^8^, ^9^, ^10^] unreactive molecules and bonds such as H_2_, CO, CO_2_, N_2_O, among others,[^7^, ^11^, ^12^, ^13^, ^14^, ^15^, ^16^, ^17^, ^18^] and the development of novel and more efficient FLP systems (even incorporating transition‐metal components).^[19]^ Additionally, extensive research has been conducted to explore mechanistic details in the field of FLPs over the past decade.[^5^, ^20^, ^21^, ^22^, ^23^, ^24^] These complexes function as effective metal‐free hydrogenation catalysts, providing a tantalizing glimpse into a new era of catalytic capability without traditional transition metals.

**Scheme 1:**
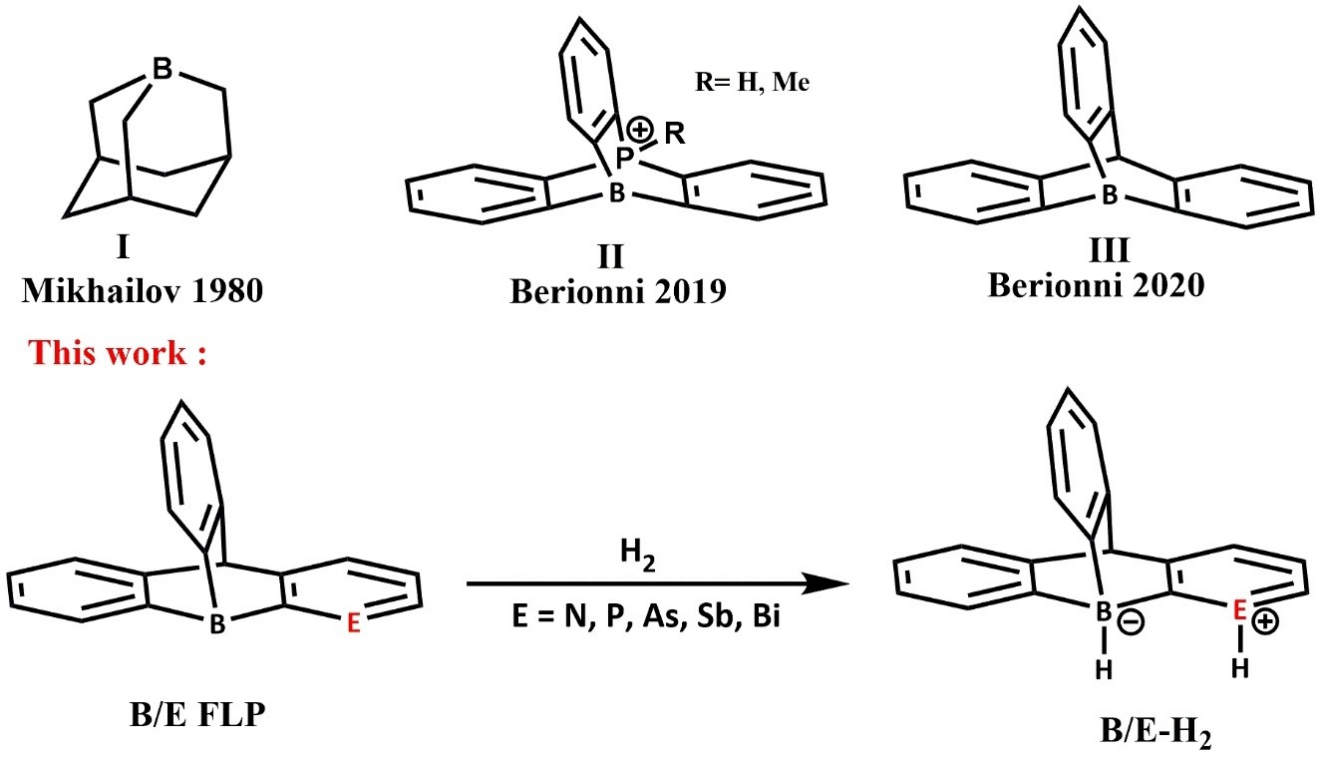
Heterolytic spitting of the hydrogen molecule by the considered FLP systems.

The catalytic system‘s effectiveness relies on the presence of sufficiently strong Lewis acids, as weak Lewis acids are not catalytically active.[^25^, ^26^, ^27^, ^28^, ^29^] Conversely, excessively strong Lewis acids lead to highly stable reaction products, inhibiting the formation of the catalytic cycle. Therefore, a key objective in research is to develop group 13 Lewis acids with adjustable Lewis acidity, which is an ongoing and important endeavor.[^30^, ^31^, ^32^, ^33^] Many studies focus on modifying the substituents on the group 13 center by incorporating perfluorinated molecules, thereby significantly enhancing the Lewis acidity.^[34]^ Lewis acids made of boron are the classic trigonal planar Lewis acids; they undergo structural remodeling upon complex formation from their planar geometry to a pyramidal structure which needs a significant amount of distortion energy. Consequently, an alternative approach to increase Lewis acidity involves the synthesis of compounds featuring a pyramidalized group 13 Lewis center.[^35^, ^36^, ^37^] Pioneered by Piers, borabarrelene and benzaborabarrelene are early instances of pyramidal Lewis acids.^[38]^ An exemplary instance of a group 13 Lewis acid with a pyramidal structure is boraadamantane (**I** in Scheme 1).^[39]^ This compound exhibits remarkable stability when it forms a molecular complex with azaadamantane, showcasing robust bonding and exceptional resilience against degradation in the presence of air.^[40]^ Systems based on stiff, rigid aromatic spacers coupled with bora‐and ala‐adamantane scaffolds were constructed and computationally investigated.^[41]^

The synthesis and DFT application of 9‐boratriptycene derivatives **II** and **III** (Scheme 1) as Lewis acids were reported by Berionni et al., demonstrating how the non‐planar structure of boron compounds significantly increases their Lewis acidity due to the reduction in their reorganization (or distortion) energies.[^42^, ^43^] More computational studies were conducted to explore their chemical reactivity after the computational validation of 9‐boratriptycene, 9‐alartriptycene, and their perfluorinated derivatives as very efficient pyramidal Lewis acids.^[36]^ This drives us to investigate the dihydrogen activation reaction of 9‐boratriptycenes in combination with a Lewis base in order to design a new metal‐free bifunctional system for dihydrogen activation. In this article, we propose **B/E FLP** systems (E=N, P, As, Sb, and Bi) with a tri‐coordinated boron center embedded in an unusual pyramidal geometry as an ideal candidate for heterolytic hydrogen molecule splitting (Scheme 1). Since derived from already isolated scaffolds (9‐boratriptycene derivatives), we perceive the considered systems as feasible and achievable through synthetic methods. The concerned reaction is computationally investigated using DFT. In order to investigate the effects of the distortion and interaction energies on the activation of the hydrogen molecule along the reaction pathway, the activation strain model (ASM) is applied to all systems, and the thermodynamics and kinetics (transition states) of the reactions are also discussed.

Among the diverse utilizations of FLPs, the reduction of unsaturated organic substrates through hydrogenation emerges as a particularly emblematic reaction.[^11^, ^44^] This method, which does not involve transition metals and can be catalytic, poses a significant challenge when it comes to achieving enantioselective hydrogenations using chiral FLPs.^[45]^ Numerous research studies have been conducted to investigate FLP‐mediated hydrogenation reactions that involve multiple bonds.^[46]^ These reactions follow a sequence where H_2_ is first activated, and then H is transferred to the substrate. The transfer of H^+^ and H^−^ to the substrate occurs through two distinct mechanisms. In the cases where strong Lewis acid (LA) components like B(C_6_F_5_)_3_ are involved, the substrate activation requires protonation or interaction through H‐bonding because the conjugate base alone lacks the necessary strength to donate H^−^. Thus, protonation takes place before the H^−^ transfer.[^47^, ^48^, ^49^] In such situations, it is common for the substrate itself to act as the Lewis base.^[49]^ Alternatively, another mechanism involves H^−^ transfer happening prior to H^+^ transfer, requiring the substrate to be activated with another Lewis acid to facilitate the hydride transfer.^[50]^ Additionally, a simultaneous transfer of H^+^ and H^−^ to the substrate, known as concerted transfer, is observed in the hydrogenation of CO_2_ to HCOOH.^[51]^ In this article, we also opted to explore the transformation further with the presence of **B/E‐FLP**. Our study primarily centered on the subsequent release of dihydrogen, which facilitates the reduction of various multiple bonds.

## Computational Details

Geometry optimization was carried out on the significant structures within the potential energy surfaces utilizing the Gaussian 16 quantum chemical programs.^[52]^ The process of dihydrogen activation by B/E FLP was investigated utilizing the meta‐hybrid M06‐2X functional in combination with the split valence polarization basis set def2‐SVP.^[53]^ This approach yields more reliable results for describing the kinetics of main group reactions. Vibrational frequency analyses were employed to characterize the desired minima and transition state geometries. The identification of transition state (TS) geometries involved visualizing the reaction coordinate by animating an imaginary frequency. Single‐point energies were computed using the polarizable continuum model (PCM) at the PCM(toluene)‐M06‐2X/def2‐TZVP//M06‐2X/def2‐SVP level of theory to incorporate the influence of solvation.[^54^, ^55^] The NBO 6.0 program was used to conduct principal interacting orbital (PIO) analysis.^[56]^ The optimized geometries were demonstrated using CYLView and ChemDraw software.^[57]^

### Activation strain model (ASM)

In order to attain a more comprehension of the activation barriers in the reaction, the ASM (Activation Strain Model) method was employed.[^58^, ^59^] This approach is based on fragments and aims to elucidate the chemical reaction by examining the activation barrier in terms of isolated reactants.[^60^, ^61^] The potential energy along the reaction coordinate Δ*E*(ζ) is determined by two primary factors: the strain (Δ*E*_strain_(ζ)) arising from the deformed reactants, and the interaction energy (Δ*E*_int_(ζ)) between the deformed reactants (Eq. 1.
(1)

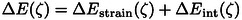




The strain Δ*E*_strain_(ζ) is influenced by two aspects: the trajectory followed along the reaction coordinate and the inherent rigidity of the reactants. In contrast, when two interacting reactants approach each other, there is a modification in the electronic configuration, which predominantly accounts for the interaction energy, denoted as Δ*E*_int_(ζ). The combined impact of Δ*E*_strain_(ζ) and Δ*E*_int_(ζ)determines the location of the barrier, specifically at the point governed by Equation 2. This approach plays a crucial role in comprehending the mechanisms of organic and organometallic reactions.

(2) $$\frac{d\Delta E_{strain}\left(\zeta \right)}{d\zeta }=-\frac{d\Delta E_{int}\left(\zeta \right)}{d\zeta }$$

### Energy decomposition analysis

To obtain the energies associated with different contributing factors to the total interaction energy Δ*E*_int_(ζ), an energy decomposition analysis (EDA) is conducted.[^62^, ^63^] Equation 3 provides the breakdown of these terms along the reaction coordinate.
(3)

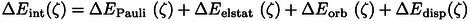




The aforementioned terms have the following interpretations: Δ*E*_elstat_ corresponds to the electrostatic interaction energy, Δ*E*_Pauli_ represents the Pauli repulsion energy, Δ*E*_orb_ signifies the orbital energy, and Δ*E*_disp_ denotes the dispersion energy. The involvement of orbital energies arising from different bond types, including sigma (σ), pi (π), and delta (δ), is computed through the utilization of natural orbitals for chemical valence within the framework of the extended transition state theory (ETS‐NOCV) technique, as outlined in Equation 4.

(4)

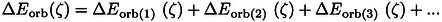




The ETS‐NOCV analysis was conducted using the B3LYP‐D3(BJ)/TZ2P//M06‐2X/def2‐SVP method,[^64^, ^65^, ^66^, ^67^, ^68^, ^69^, ^70^] utilizing the Amsterdam Density Functional (ADF 2013.01) package.

### Conceptual density functional theory (CDFT)

The concept of electronegativity, originally proposed by Pauling,^[71]^ refers to an atom‘s capability to attract a pair of electrons in a chemical bond. On the other hand, Pearson introduced the concept of hardness.^[72]^ The wave function and electronic energy of a system are influenced by both the external potential $v\left(\vec{r}\right)$ and the number of electrons, N. The system‘s first‐order response to changes in N is characterized by the electronegativity (*χ*) and chemical potential (*μ*), which have equal magnitudes but opposite signs.^[73]^ Hardness, on the other hand, represents the system‘s second‐order response to changes in N, while $v\left(\vec{r}\right)\;$ remains constant.^[74]^

(5) $$\chi =-\mu ={-\left(\frac{\partial E}{\partial N}\right)}_{v\left(\vec{r}\right)\;}$$

(6) $$\eta ={\left(\frac{\partial^{2}E}{\partial N^{2}}\right)}_{v\left(\vec{r}\right)\;}={\left(\frac{\partial \mu }{\partial N}\right)}_{v\left(\vec{r}\right)\;}$$

As per the finite difference method,^[75]^ the definitions of hardness and electronegativity can be stated as follows:

(7) η≅I-A

(8) $$\chi =\frac{I+A}{2}$$

The first ionization potential (*I*) corresponds to the minimum energy necessary for the removal of the outermost electron from the nucleus of a species in its gaseous phase. Conversely, electron affinity (*A*) designates the energy released upon the addition of an electron to the outermost orbital of a species. Electrophilicity (*ω*), as defined by Parr et al.,^[76]^ is another term used to describe reactivity.[^77^, ^78^, ^79^]

(9) $$\omega =\frac{\mu^{2}}{2\eta }=\frac{\chi^{2}}{2\eta }$$

The determination of *I* and *A* values involves the use of the ΔSCF (Difference in Self‐Consistent Field) method,^[75]^ which entails calculating the single‐point energies of the N‐electron system, its cation (N ‐ 1), and anion (N+1.

(10) $$I=E_{N-1}-E_{N}$$

(11) $$A=E_{N}-E_{N+1}$$

By applying the principles of Fukui's frontier orbital theory, the reactivity and selectivity of a particular local site can be assessed through the utilization of Fukui functions.^[80]^ These functions play a pivotal role in determining the reactivity and selectivity of chemical species at that specific site.

(12) $$f\left(\vec{r}\right)={\left(\frac{\partial \rho \left(\vec{r}\right)}{\partial N}\right)}_{v\left(\vec{r}\right)}={\left(\frac{\partial \rho \left(\vec{r}\right)}{\partial v\left(\vec{r}\right)}\right)}_{N}$$

Three Fukui functions are derived by examining the discontinuities of $\rho \left(\vec{r}\right)$ with respect to changes in the number of electrons (N):^[81]^

(13a) $$\mathrm{attack}\;\mathrm{of}\;a\;\mathrm{nucleophile}f^{+}\left(\vec{r}\right)=\rho_{N+1}\left(\vec{r}\right)-\rho_{N}\left(\vec{r}\right)\;$$

(13b) $$\mathrm{attack}\;\mathrm{of}\;\mathrm{an}\;\mathrm{electrophile}f^{-}\left(\vec{r}\right)=\rho_{N}\left(\vec{r}\right)-\rho_{N-1}\left(\vec{r}\right)\;$$

(13c) $$\mathrm{for}\;\mathrm{radical}\;\mathrm{attack}f^{0}\left(\vec{r}\right)={\frac{1}{2}[\rho }_{N+1}\left(\vec{r}\right)-\rho_{N-1}\left(\vec{r}\right)]\;$$

$\rho_{M}\left(\vec{r}\right)$ represents the electron density at a specific $v\left(\vec{r}\right)$ for a species with M electrons. By replacing the electron population ($q_{k}$ ) for the electron density $\rho \left(\vec{r}\right)$ of a molecule, the corresponding condensed Fukui functions at the atomic site k can be obtained.

(14) $$f_{k}^{\alpha }\;;\;\alpha =0,\;-,\;\mathrm{and}\;+$$

In the given context, where α equals to 0, +, and ‐ represent radical, nucleophilic, and electrophilic attacks respectively. The normalization of the Fukui function applies uniformly across the entire molecule. This normalization leads to the following expression:

(15) $$\int f\left(\vec{r}\right)dr=1$$

The depiction of the electrophilicity at a specific atomic position within a species is illustrated utilizing the resolution of identity as:[^82^, ^83^]

(16) $$\omega \left(\vec{r}\right)=\omega f\left(\vec{r}\right)$$

Hence, three different local electrophilicities denoted as $\omega^{\alpha }\left(\vec{r}\right)$ are derived based on the information of *ω* and $f\left(\vec{r}\right)$ as:

(17) $$\omega^{\alpha }\left(\vec{r}\right)=\omega f^{\alpha }\left(\vec{r}\right)$$

The representation of an atomic site *k* within a molecule as a condensed‐to‐atom expression can be formulated as follows:

(18) $$\omega_{k}^{\alpha }=\;\omega f_{k}^{\alpha }$$

When α is equal to 0, positive or negative, it represents the radical, nucleophilic, and electrophilic attacks, respectively.[^84^, ^85^]

## Results and Discussion

### Dihydrogen activation

**Figure 1:**
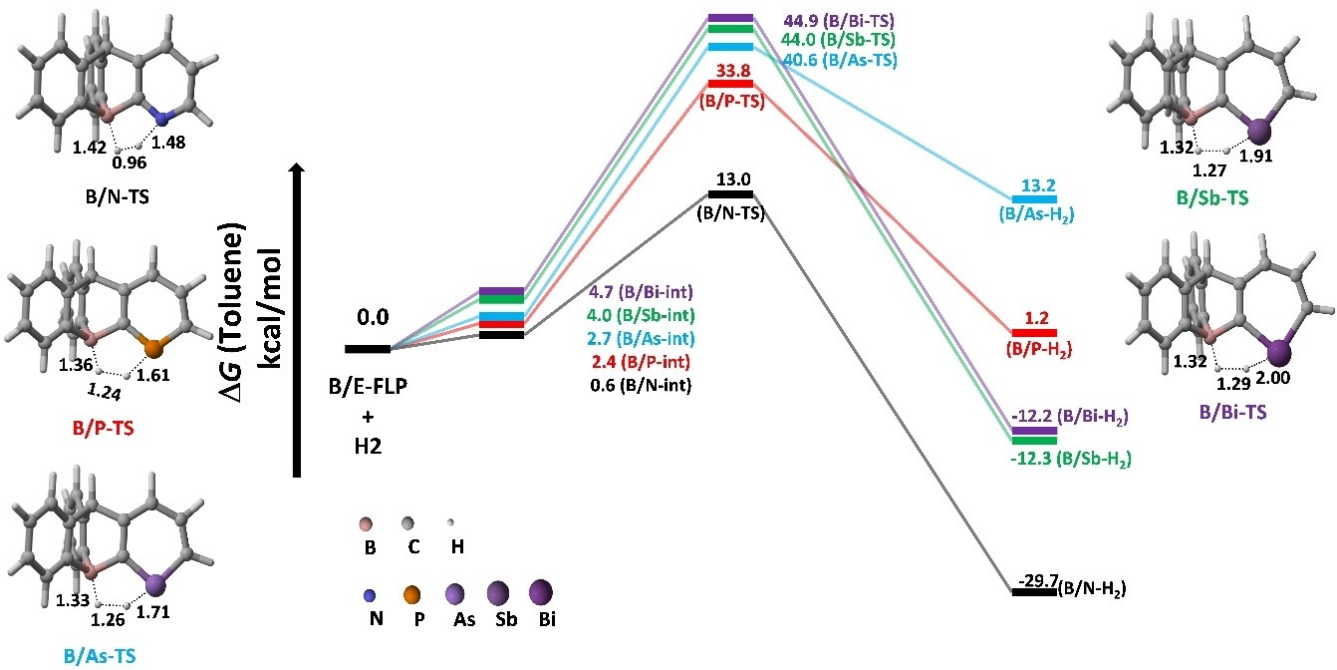
Solvent corrected relative free energy profile for the dihydrogen activation reaction by B/E‐FLP.

The dihydrogen activation mechanism was examined using the **B/E‐FLP** type molecules, as shown in Scheme 1. Here **E** represents a group 15 element, namely N, P, As, Sb or Bi. The computed free energy of activation (ΔG^≠^) and reaction free energies (ΔG_R_), as well as the geometries of **B/E‐TS** with requisite bond lengths, are shown in Figure 1.

Our DFT findings demonstrated that the dihydrogen activation reaction performed by a **B/E‐FLP** takes place in a concerted manner via a five‐membered transition state (**B/E‐TS**), culminating in the formation of a zwitterionic adduct (**B/E‐H_2_**). The activation free energy (ΔG≠) at 298.15 K, measured in kcal/mol, exhibits an ascending trend (see Table 1) as follows: **B/N‐TS** (12.4)<**B/P‐TS** (31.4)<**B/As‐TS** (37.9)<**B/Sb‐TS** (40.0)<**B/Bi‐TS** (40.2). Moreover, the reaction free energies (ΔG_R_) (kcal/mol) for the dihydrogen activation process were estimated as follows **B/N−H_2_** (−29.7)<**B/Sb‐H_2_** (−12.3)<**B/Bi‐H_2_** (−12.2)<**B/P‐H_2_** (1.2)<**B/As‐H_2_** (13.2). From Figure 1, the H−H distance at the TSs follows the order d_H−H_ (0.96 Å in **B/N‐TS**)<d_H−H_ (1.24 Å in **B/P‐TS**)<d_H−H_ (1.26 Å in **B/As‐TS**)<d_H−H_ (1.27 Å in **B/Sb‐TS**))<d_H−H_ (1.29 Å in **B/Bi‐TS**) as well as the E−H distance in the TSs also follows the order d_N−H_ (1.48 Å in **B/N‐TS**)<d_P−H_ (1.61 Å in **B/P‐TS**)<d_As−H_ (1.71 Å in **B/As‐TS**)<d_Sb−H_ (1.91 Å in **B/Sb‐TS**)<d_Bi−H_ (2.00 Å in **B/Bi‐TS**). The order of the previous trends aligns with the activation free energy sequence. The activation barrier trends observed in dihydrogen activation by the **B/E‐FLP** system are possibly attributed to the heavier atomic number of the **E** center in the **B/E FLP**‐type molecule. Theoretical findings suggest that as the atomic radius (pm) of the **E** center increases, the activation barrier also increases. The atomic radius sequence of the **E** center is as follows: N (71)<P (107)<As (119)<Sb (139)<Bi (148).^[86]^ Based on the kinetic and thermodynamic data mentioned above, the **B/N‐FLP** system exhibits the lowest activation barrier.

**Table 1:** Solvent corrected reaction free energies for the dihydrogen activation reaction by B/E‐FLP in kcal/mol.

System	Reactants	Intermediate	TS	Activation Energy(Δ*G* ^≠^)	Product
**B/N‐FLP+H_2_**	0.0	0.6	13.0	12.4	−29.7
**B/P‐FLP+H_2_**	0.0	2.4	33.8	31.4	1.2
**B/As‐FLP+H_2_**	0.0	2.7	40.6	37.9	13.2
**B/Sb‐FLP+H_2_**	0.0	4.0	44.0	40.0	−12.3
**B/Bi‐FLP+H_2_**	0.0	4.7	44.9	40.2	−12.2

**Figure 2:**
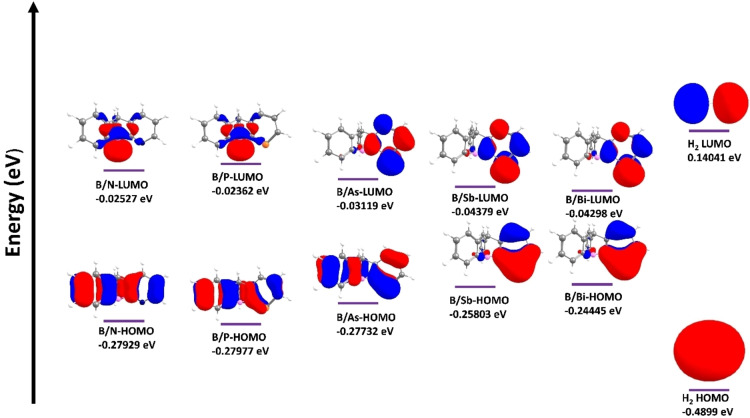
Selected FMOs (HOMO and LUMO) and their energies (in eV) of the B/E FLPs and dihydrogen.

In order to better understand the factors contributing to the barriers in the H_2_ activation reaction by **B/E‐FLP**, we conducted an analysis of their electronic structures using frontier molecular orbitals (FMOs). Figure 2 illustrates the shapes of the highest occupied molecular orbital (HOMO) and lowest unoccupied molecular orbital (LUMO) for the **B/E‐FLP**, along with corresponding energy values (eV). It is evident that the HOMO of **B/E‐FLP** predominantly resides at the Lewis basic E element, while the LUMO is primarily located at the Lewis acidic B center. To further examine the energetic aspects, Table 2 provides the energy differences between the LUMO of H_2_ and the HOMO of **B/E‐FLP**, as well as between the LUMO of **B/E‐FLP** and the HOMO of H_2,_ utilizing the data from Figure 2. In all cases, the calculated energy differences for the former (ranging from 0.38 to 0.43 eV) were considerably smaller compared to those for the latter (ranging from 0.44 to 0.47 eV). The theoretical deductions strongly indicate that the primary interaction in each scenario is likely between the HOMO of the **B/E‐FLP** system and the LUMO of dihydrogen.

**Table 2:** Energy difference (in eV) between the FMOs of the B/E FLPs and dihydrogen.

System	Energy difference H_2_(LUMO) − FLP(HOMO)	Energy difference FLP(LUMO)‐H_2_(HOMO)
**B/N‐FLP+H_2_**	0.419	0.465
**B/P‐FLP+H_2_**	0.420	0.466
**B/As‐FLP+H_2_**	0.418	0.459
**B/Sb‐FLP+H_2_**	0.398	0.446
**B/Bi‐FLP+H_2_**	0.385	0.447

**Figure 3:**
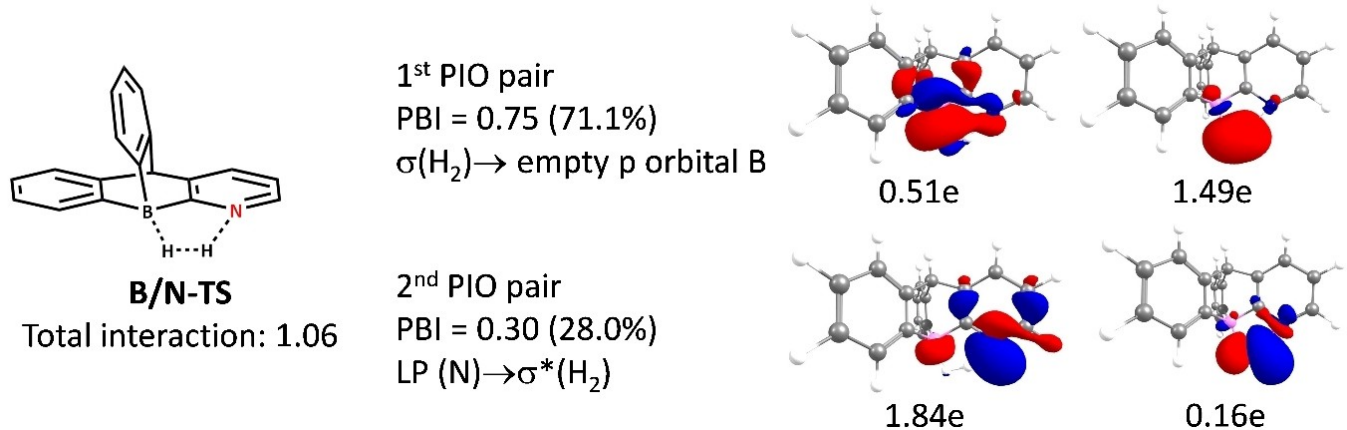
PIO analysis of B/N‐TS using B/N‐FLP and H_2_ as two fragments. PBI and its percentage contribution to the overall interactions between the two are also shown. The isovalue is 0.03.

The Lin Group has introduced a concept called Principal Interacting Orbital (PIO) analysis,[^87^, ^88^] which offers an intuitive approach to identifying and quantifying bonding interactions between two fragments. This analysis provides valuable insights into the chemistry involved. The bonding strength between the associated PIO pairs can be described using a parameter known as the PIO‐based Bond Index (PBI). The PBI encapsulates the intensity of interaction between the two fragments. Figure 3 depicts the decomposition of **B/N‐TS** into two fragments: the H_2_ moiety and the B/N‐FLP moiety. The primary interactions are clearly depicted. The first PIO pair of **B/N‐TS** involves the interaction between the empty p‐orbital of the B center and the σ bond of H_2_, yielding a PBI value of 0.75. The σ bond of H_2_ contributes 1.49e to the interaction, while the boron center provides 0.51e. Moving on to the second PIO pair of B/N‐TS, it shows that the lone pair of electrons from the nitrogen center is donated to the σ* orbital of H_2_. This donation leads to a PBI value of 0.30, with the nitrogen center contributing 1.84e and the antibonding orbital of H_2_ providing 0.16e to this interaction. The PIO analysis of other respective TSs are shown in Figure S1. The structural reorganization and interactions involving specific orbitals, such as the lone pair of **E** center, the bonding and antibonding orbitals of H_2_, and the empty p orbital of boron at the transition state, facilitate the breaking of the H−H bond. This supports Papai's Electron Transfer (ET) model,^[89]^ which emphasizes the involvement of electron transfer in the reaction mechanism.

**Figure 4:**
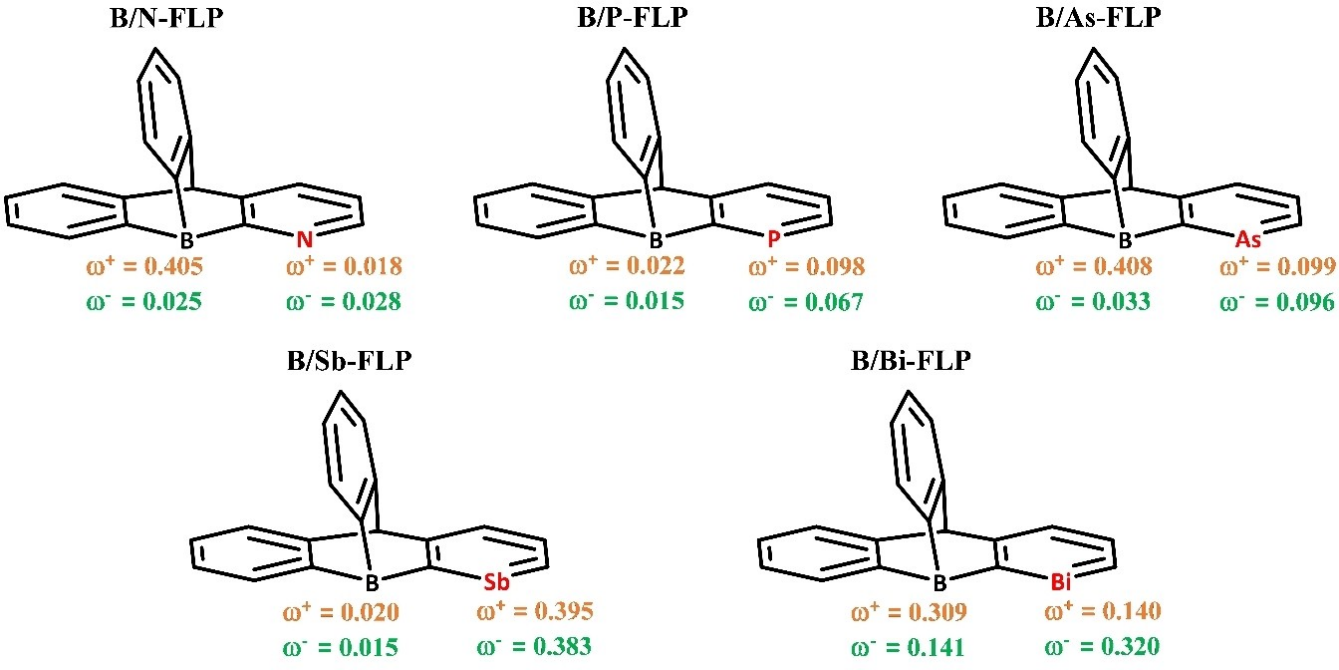
Local electrophilicity indices ω^+^_k_ (orange) and ω^−^_k_ (green) on the concerned reactive center of the FLPs.

The electrophilicity of the reactive atoms in **B/E‐FLP** systems has been calculated and pictorially depicted in Figure 4. Based on the obtained data, in the **B/N‐FLP** system, the ω^−^_k_ (green) value (0.028) for the N center is higher than the ω^+^_k_ (orange) value (0.018). This suggests that the N center is more likely to act as a nucleophilic center or be susceptible to electrophilic attack.[^90^, ^91^, ^92^] Conversely, for the B center, the ω^+^_k_ (orange) value (0.405) is higher than the ω^+^_k_ (green) value (0.025), suggesting that the B center can exhibit either electrophilic behavior or susceptibility to nucleophilic attack. Similarly, in all the examined B/E‐FLP, the corresponding B centers will act as electrophilic centers. The values of ω^+^_B_ and ω^−^_LA_ are graphed against the activation barrier of the corresponding FLPs, as depicted in Figure S2. A good correlation is observed in both instances. Consequently, the CDFT findings provide additional support for the validity of the reactivity trends.

**Figure 5:**
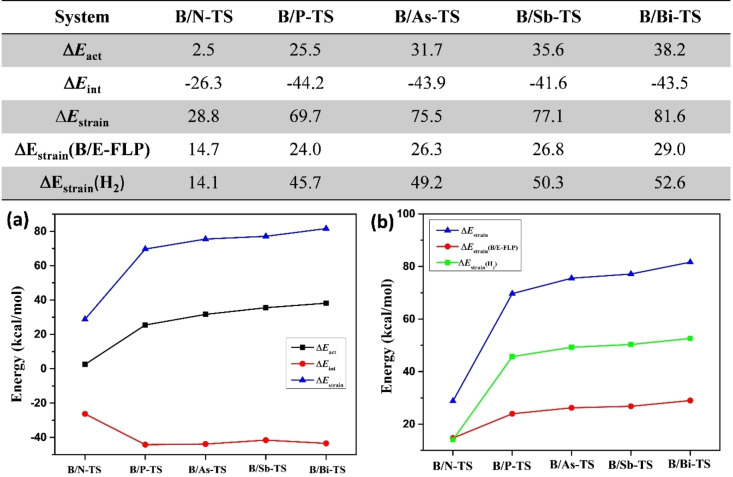
Energy decompositions of the activation energies (ΔE_act_) of the transition states for the dihydrogen activation reactions.

In order to gain a deeper understanding of the primary factor impacting the activation barrier during the dihydrogen activation process, we employed the ASM model to assess the transition state barrier with respect to the isolated reactants. The activation energy was deconstructed into two components: the interaction energy between the two fragments (B/E‐FLP and H_2_) and their individual strain energy. These findings are visually represented in Figure 5. Interestingly, the interaction energy (**Δ*E***_**int**_) does not exhibit the same pattern as the activation energy, while the strain energy (**Δ*E***_**strain**_) appears to be the crucial factor governing the variation in the activation barrier. Analysis of the data presented in Figure 5 reveals that the strain energy of the respective system follows the order: **Δ*E***_**strain**_(**B/N‐TS**)<**Δ*E***_**strain**_(**B/P‐TS**)<**Δ*E***_**strain**_(**B/As‐TS**)<**Δ*E***_**strain**_(**B/Sb‐TS**)<**Δ*E***_**strain**_(**B/Bi‐TS**). Additionally, by decomposing the strain energy into two fragments, namely H_2_ and the remaining components, it becomes evident that the destabilizing strain energy increases from **B/N‐FLP** to **B/Bi‐FLP**. Similarly, the strain energy of dihydrogen also increases in line with the activation energy barrier. Consequently, both of these factors significantly contribute to the activation barrier of the reaction. From the ASM study, it also concludes that B/N‐FLP has the least activation barrier for dihydrogen activation.

### Hydrogenation of multiple bonds

**Scheme 2:**
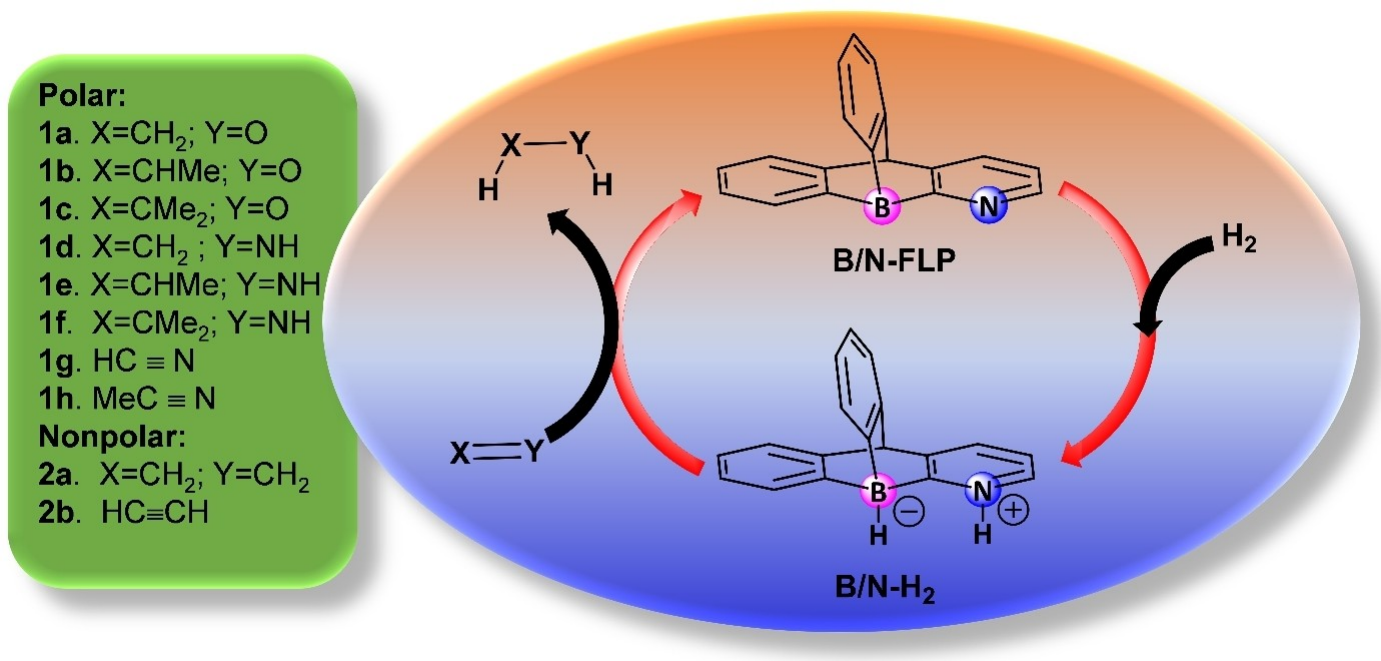
Hydrogenation of multiple bonds using the B/N‐FLP system.

Due to its minimal activation barrier, we opted to explore the transformation further with the presence of **B/N‐FLP**. Our study primarily centered on the subsequent release of dihydrogen, which facilitates the reduction of various multiple bonds. Specifically, we focused on processes involving different types of multiple bonds, namely polar bonds such as O=C, N=C, and C≡N, as well as non‐polar bonds like C=C and C≡C (Scheme 2).

**Figure 6:**
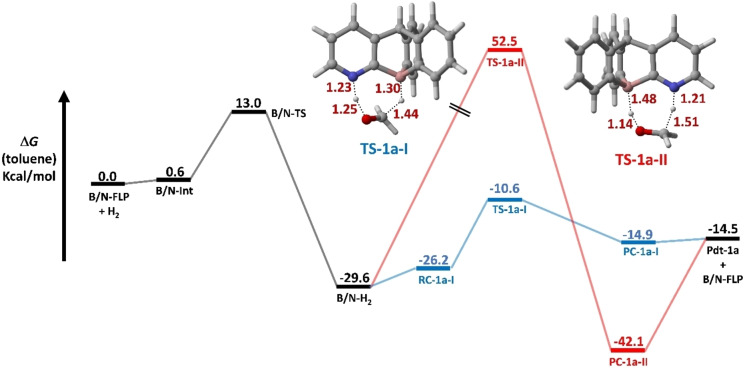
Computed solvent corrected relative free profile for the hydrogenation reaction of formaldehyde (**1 a**) by B/N‐FLP. Relative free energies (ΔG, computed at 298 K) in kcal/mol. Bond distances are provided in Å.

Figure 6 illustrates the computed reaction profile for a hydrogenation reaction involving the activation of dihydrogen by B/N‐FLP and subsequent reduction of formaldehyde (**1 a**). The reduction process of substrate **1 a** by B/N−H_2_ occurs through two different pathways. Pathway‐I involves a concerted approach initiated by the reactant complex **RC‐1 a‐I**. This complex then transforms into the corresponding reaction products via the transition state **TS‐1 a‐I** with an activation barrier (ΔG^≠^) of 15.6 kcal/mol, leading to the formation of the product complex **PC‐1 a‐I** followed by the formation of methanol (CH_3_−OH) and B/N‐FLP. The transition state (**TS‐1 a‐I**) is characterized by the concerted (yet asynchronous) migration of both hydrogen atoms from the zwitterionic (**B/N−H_2_**) species to formaldehyde (**1 a**). This migration entails the nucleophilic attack of the B−H hydride species on the electrophilic carbon atom of **1 a**, as well as the N−H proton migration onto the oxygen atom of **1 a**. This resemblance is akin to familiar reactions, such as diimide reduction, employed for double or triple bonds, as well as the Meerwein‐Ponndorf‐Verley reduction (MPV) applied to carbonyl groups. Furthermore, it bears similarity to certain type II dyotropic reactions, which involve the internal transfer of two groups, often hydrogen atoms. Additionally, this conceptualization draws parallels to transition‐metal‐catalyzed hydrogenation reactions of polar bonds, as seen in the Noyori‐type hydrogenation.

Alternatively, in another pathway, the formation of the reaction products can occur via a different transition state (**TS‐1 a‐II**), wherein the B−H hydride species moves towards the nucleophilic oxygen atom of the carbonyl instead of the electrophilic carbon center. It is worth noting that this unconventional reaction exhibits a significantly elevated activation barrier (ΔG^≠^=82.1 kcal/mol), making this alternate pathway impractical from a kinetic standpoint. Furthermore, incorporating methyl groups onto the carbon atom within the formaldehyde substrate (**1 a**) resulted in higher energy barriers (ΔG^≠^ (kcal/mol) increased in the order **1 a** (15.6)<**1b** (19.3)<**1c** (23.6), indicating that steric hindrance at that specific carbon atom hinders the nucleophilic attack of the B−H hydride species (see Figure S4). However, the energy profile (Figure S3 in the supporting information) illustrates the solvent‐corrected relative free energies using toluene, cyclohexane, dichloromethane (DCM), and dimethyl sulfoxide (DMSO) as solvents. Analyzing this energy profile reveals significant insights into the activation barriers for dihydrogen activation in non‐polar solvents like toluene and cyclohexane, viz., 12.4 kcal/mol and 12.2 kcal/mol, respectively. In contrast, for polar solvents such as DCM and DMSO, these barriers slightly increase to 13.3 kcal/mol and 13.7 kcal/mol, respectively. Notably, the exothermicity of the dihydrogen reaction shows a slight increase in polar solvents. Examining the subsequent energy profile step, the reduction of formaldehyde proceeds via two pathways. Pathway I proceeds via **TS‐1 a‐I**, exhibiting activation barriers of 15.6 kcal/mol (in toluene), 15.4 kcal/mol (in cyclohexane), 17.0 kcal/mol (in DCM), and 16.6 kcal/mol (in DMSO). However, for Pathway II, the reaction progresses via transition state **TS‐1 a‐II**, displaying notably higher activation barriers of 82.1 kcal/mol (in toluene), 81.6 kcal/mol (in cyclohexane), 86.1 kcal/mol (in DCM), and 85.0 kcal/mol (in DMSO). It is evident that the activation barrier slightly increases in polar solvents for both pathways.

In contrast, a systematic observation revealed that the hydrogenation of methanimine (**1 d**) and hydrogen cyanide (**1 g**) presented greater challenges in terms of kinetics compared to the hydrogenation of formaldehyde (**1 a**). The activation barrier (depicted in Figure S5a) for the hydrogenation of **1 d** is 17.9 kcal/mol (ΔG^≠^) (in the case of pathway I), which is approximately 2.3 kcal/mol higher than the activation barrier for the hydrogenation of formaldehyde. Similarly, the activation barrier (shown in Figure S6a) for the hydrogenation of **1 g** is 35.8 kcal/mol (ΔG^≠^). This observation corresponds to the lower electrophilicity exhibited by the carbon atom in substrates **1 d** and **1 g**, respectively, when compared to the electrophilicity of the carbon atom in **1 a**. On the other hand, the introduction of a methyl group to the substrate resulted in an increased activation energy barrier due to steric hindrance at the carbon atom of the respective substrates.

**Figure 7:**
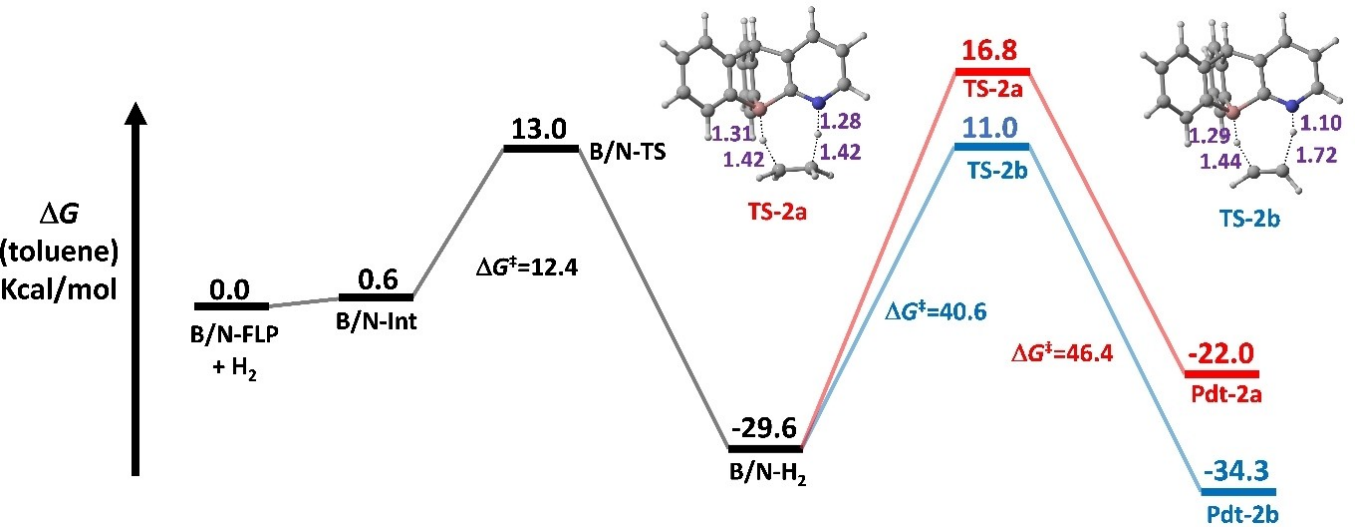
Computed solvent corrected relative free profile for the hydrogenation reaction of ethylene (**2 a**) and acetylene (**2 b**) by B/N‐FLP.

Moreover, it was discovered that the process of hydrogenating non‐polar bonds (as seen in the reactions involving substrates **2 a** and **2 b**) proceeded (depicted in Figure 7) with significantly higher activation energy barriers (ΔG^≠^=40–47 kcal/mol) compared to the similar reactions involving polar bonds. The hydrogenation process of ethylene (**2 a**) requires a higher activation energy barrier (ΔG^≠^) of 46.4 kcal/mol compared to acetylene (**2 b**), which has a lower barrier of 40.6 kcal/mol. This disparity is attributed to the nucleophilic attack of the B−H hydride to the higher electronegativity and electrophilicity of the sp‐hybridized carbon center in acetylene, as opposed to the sp^2^‐hybridized carbon center in ethylene. Consequently, the sp‐hybridized carbon center in acetylene facilitates the reaction with reduced activation energy.

**Figure 8:**
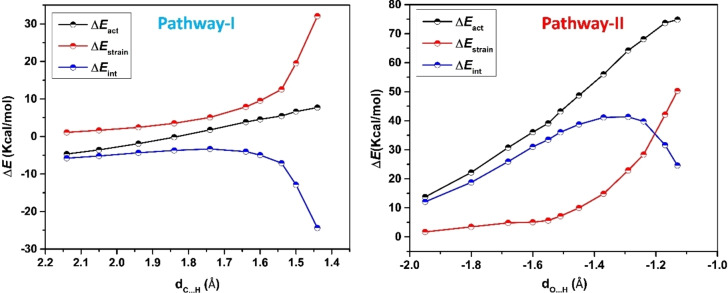
Comparison of activation‐strain diagrams illustrating hydrogenation reactions facilitated by B/N−H_2_ for pathway‐I and pathway‐II along the IRC projected onto the C…H (pathway‐I) and O…H (pathway‐II) bond‐forming distance.

The Activation Strain Model (ASM) analysis is conducted with the aim of gaining deeper insights into reactivity patterns. In this study, the ASMs are employed to examine the distances involved in the formation of C⋅⋅⋅H bonds (pathway‐I) and O⋅⋅⋅H bonds (pathway‐II) along the Intrinsic Reaction Coordinate (IRC) leading to the transition states (TSs) of the hydrogenation reaction of formaldehyde (**1 a**), mediated by the B/N−H_2_ FLP, as depicted in Figure 8. Figure 8 provides valuable data, revealing the stabilizing interaction energies (**Δ*E***_**int**_) between the reactants that have undergone distortion throughout the reaction for pathway‐I. These interaction energies reach their highest point (stabilizing energy) in close proximity to the transition state, owing to the nucleophilic attack on the electrophilic carbon atom of formaldehyde by the B−H hydride species. Conversely, for pathway‐II, the **Δ*E***_**int**_ values increase initially and become less positive at the TS stage, indicating a less favorable nature for this pathway as the B−H hydride species moves towards the nucleophilic oxygen atom of the carbonyl. Simultaneously, the term representing destabilizing strain energy (**Δ*E***_**strain**_), which signifies the energy required to deform the reactants, increases progressively as the reaction proceeds in both pathways. Consequently, the ASM methodology demonstrates that the stronger interaction energy is capable of offsetting the destabilizing strain energy term, particularly in pathway‐I, thereby lowering the activation barrier of the reaction.

Finally, we incorporate the EDA results obtained at the transition states (TSs), as presented in Table 3, by treating the respective substrate as one fragment and the rest as the other. The electrostatic interaction energy (**Δ*E***_**elstat**_) accounts for approximately 30–40 % of the overall attractive interaction energy (**Δ*E***_**int**_), as observed in the range of values. Conversely, the orbital interaction energy (**Δ*E***_**orb**_) contributes around 57–68 % to the total attractive interaction energy. Additionally, the dispersion interaction energy (**Δ*E***_**disp**_) demonstrates a modest influence, ranging from 2–4 % of the total attractive interaction energy. For the hydrogenation reaction of **1 a** by **B/N−H_2_** via two different pathways, pathway‐I exhibits a remarkably stabilizing interaction energy of −27.8 kcal/mol.

**Table 3:** EDA results for the reactions at the TSs employing the B3LYP(D3BJ)/TZ2P//M06‐2X/def2‐SVP level of theory. All energy values are expressed in kcal/mol.

System	Pathway	Δ*E*_int_	Δ*E*_Pauli_	Δ*E*_disp_^[a]^	Δ*E*_elstat_^[a]^	Δ*E*_orb_^[a]^	Δ*E*_orb(1)_	Δ*E*_orb(2)_	Δ*E*_orb(rest)_
TS‐1a	I	−27.8	193.7	−5.6(2.5)	−87.5(39.5)	−128.3(58.0)	−72.2	−37.4	−18.7
	II	16.1	260.8	−3.9(1.6)	−73.8(30.1)	−167.1(68.3)	−129.3	−27.1	−10.6
TS‐1b	I	−33.6	202.2	−6.9(2.9)	−93.8(39.7)	−135.2(57.3)	−68.2	−45.8	−21.2
	II	18.5	267.2	−5.6(2.3)	−75.5(30.4)	−167.5(67.4)	−129.0	−27.1	−11.4
TS‐1c	I	−39.5	207.8	−9.2(3.7)	−95.8(38.7)	−142.3(57.6)	−74.0	−44.5	−23.8
	II	20.4	272.9	−6.4(2.5)	−77.1(30.6)	−168.9(66.9)	−129.6	−27.5	−11.8
TS‐1d	I	−49.2	188.1	−5.7(2.4)	−85.6(36.1)	−145.9(61.5)	−84.0	−37.6	−24.2
	II	7.8	242.9	−5.5(2.4)	−76.6(32.6)	−152.9(65.1)	−114.6	−24.4	−13.9
TS‐1e	I	−64.8	202.6	−7.9(3.0)	−91.4(34.2)	−168.2(62.9)	−106.3	−32.2	−29.6
	II	10.1	247.1	−6.4(2.7)	−76.7(32.4)	−153.9(64.9)	−115.4	−24.5	−14.0
TS‐1f	I	−84.7	231.8	−9.2(2.9)	−106.3(33.6)	−201.0(63.5)	−126.3	−37.9	−36.8
	II	10.1	250.5	−8.1(3.4)	−76.5(31.8)	−155.9(64.8)	−117.2	−23.9	−14.7
TS‐1g	I	−32.4	244.5	−4.6(1.7)	−86.1(31.1)	−186.1(67.2)	−129.3	−33.4	−23.3
	II	1.1	216.3	−4.6(2.1)	−68.4(31.8)	−142.2(66.1)	−112.6	−20.8	−8.8
TS‐1 h	I	−40.3	244.3	−5.8(2.0)	−90.0(31.6)	−188.8(66.3)	−129.3	−33.2	−26.3
	II	4.1	207.5	−5.1(2.5)	−65.4(32.2)	−132.9(65.3)	−104.6	−20.1	−8.2
TS‐2a		−10.1	128.4	−5.2(3.7)	−48.4(35.0)	−84.9(61.3)	−65.5	−14.9	−4.4
TS‐2b		−12.2	180.9	−5.7(3.0)	−58.3(30.2)	−129.1(66.8)	−96.9	−22.4	−9.8

[a] The values in parentheses are percentage contributions toward the total attraction, Δ*E*_elstat_+Δ*E*_orb_+Δ*E*_disp_.

In contrast, pathway‐II shows a lower interaction energy of only 16.1 kcal/mol. The significant disparity between these energy values highlights the considerable difference, clearly favouring pathway‐I as the more favourable option. This recurring pattern consistently reinforces the notion that pathway‐I is superior, providing a more favourable course of action. In the case of pathway‐II, the positive interaction energy arises from a higher Pauli repulsion energy (**Δ*E***_**pauli**_=260.8 kcal/mol). This term outweighs the other attractive terms, resulting in a positive interaction energy. The positive interaction energy occurs when the B−H hydride species (which carries a negative charge) approaches the nucleophilic oxygen atom of the carbonyl, leading to a destabilizing interaction. Furthermore, upon examining various substrates, it becomes evident that pathway‐I consistently exhibits a remarkable stabilizing interaction energy (**Δ*E***_**int**_) compared to pathway‐II, as shown in Table 3.

**Figure 9:**
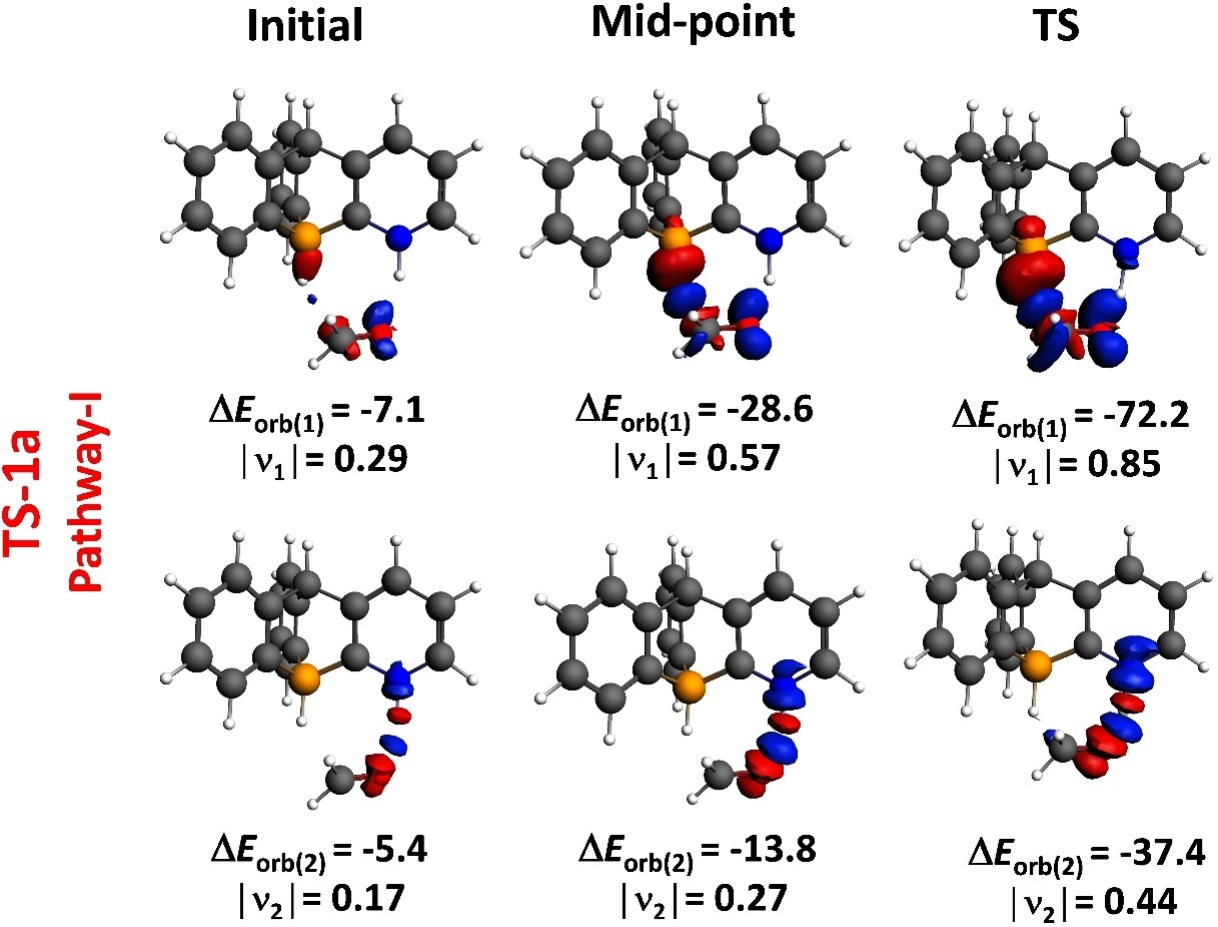
The configuration of deformation density Δρ(1) and Δρ(2) is connected with the interaction of orbitals ΔE_orb(1)_ and ΔE_orb(2)_ within the transition states, along with the eigenvalues |νn|. The flow of charge is illustrated with colors transitioning from red to blue. (isosurface value =0.003).

The investigation of the orbital interactions between the distorted reactants along the path of the reaction demands significant focus. Therefore, we decided to utilize the energy decomposition analysis combined with the natural orbitals for chemical valence (EDA‐NOCV) methodology. Through this approach, we are not only able to pinpoint but also to measure the key orbital interactions taking place between the reactants throughout the transformation. Within the framework of the EDA‐NOCV technique, two pivotal pairwise orbital interactions emerge as dominant factors in the **Δ*E***_**orb**_ term: σ(B−H) (**B/N−H_2_**)→π*(O=CH_2_‐formaldehyde) and the LP (O‐formaldehyde)→σ*(N−H) (**B/N−H_2_**) interactions. In order to visually represent these interactions at critical stages during the progression of the reaction (specifically, at the start, middle, and transition state), we display snapshots of the corresponding deformation densities of NOCV in Figure 9, labeled as **Δ*E***_**orb(1)**_ and **Δ*E***_**orb(2)**_. Initially, both interactions come into play. The calculated NOCV‐orbital energy values (**Δ*E***_**orb(1)**_) reveal that this process is consistently reinforced as the reaction advances (with **Δ*E***_**orb(1)**_ rising from −7.1 kcal/mol in the initial complex to −72.2 kcal/mol at the transition state). Notably, it is within the vicinity of the transition state that this orbital interaction becomes notably influential, playing a significant role in the overall orbital attractions between the distorted reactants. Simultaneously, the other term (**Δ*E***_**orb(2)**_), mainly associated with the migration of protic hydrogen from B/N−H_2_ to the O atom of formaldehyde, also exhibits an increase from the initial complex to the vicinity of the transition state, thus providing firm evidence of the substantial asynchronicity in this hydrogen transfer reaction. This co‐operative mechanism bears a striking resemblance to the characteristic behaviour observed in Lewis acid‐catalyzed hydride nucleophilic addition to multiple bonds. Figure S7 in the supplementary information illustrates the pairwise orbital interaction involved in the hydrogenation of the other substrates using the **B/N−H_2_**.

## Conclusions

The activation of dihydrogen using **B/E‐type** frustrated Lewis pairs (FLPs) occurs in a concerted manner, leading to the heterolytic cleavage of dihydrogen. The activation energy barrier for this process varies depending on the “**E**” center involved, and it has been established that the **B/N‐FLP** system exhibits the lowest barrier among the studied systems. This observation aligns with the activation strain model, which indicates an increase in destabilizing strain energy from **B/N‐FLP** to **B/Bi‐FLP**, supporting the trend in activation barriers. It′s of note that the energy barrier tied to H_2_ activation by FLPs exhibits a strong correlation with the co‐operative nature of these orbital interactions, namely, the fact that σ(H_2_)→ empty p orbital of B and LP(E)→σ^*^(H_2_). The liberation of activated dihydrogen by B/N‐FLP, leading to the reduction of unsaturated bonds, transpires in a concerted yet notably asynchronous fashion, akin to classical reactions involving double group transfers. This bears a resemblance to reactions like diimide reduction of double and triple bonds. Hydrogenating polar multiple bonds (such as C=O, C=N, and C≡N) is inherently more kinetically favorable compared to the corresponding hydrogenation process involving non‐polar bonds (like C=C, C≡C). According to the EDA‐NOCV method for formaldehyde reduction process, during this process, two primary orbital interactions prominently control the overall orbital attractions between the reactants: σ(B−H) (**B/N−H_2_**)→π*(O=CH_2_‐formaldehyde) and the LP (O‐formaldehyde)→σ*(N−H) (**B/N−H_2_**) interactions. Finally, this specific hydrogenation reaction can be understood as a co‐operative process involving the simultaneous migration of two hydrogen atoms. In this scenario, the initial transfer of a hydride from B−H triggers the polarization of the multiple bonds, creating the conditions necessary for the subsequent movement of the protic N−H unit.

## Conflict of interests

The authors declare no conflict of interest.

1

## Supporting information

As a service to our authors and readers, this journal provides supporting information supplied by the authors. Such materials are peer reviewed and may be re‐organized for online delivery, but are not copy‐edited or typeset. Technical support issues arising from supporting information (other than missing files) should be addressed to the authors.

Supporting Information

## Data Availability

The data that support the findings of this study are available from the corresponding author upon reasonable request.

## References

[1] R. Noyori , S. Hashiguchi , Acc. Chem. Res. 1997, 30, 97–102.

[2] M. Ito , T. Ikariya , Chem. Commun. 2007, 5134. 10.1039/b709704b 18060121

[3] B. J. Lemon , J. W. Peters , Handbook of Metalloproteins, Vol. 2 *(Eds.*: *)*, Wiley, New York, 2001.

[4] G. C. Welch , R. R. S. Juan , J. D. Masuda , D. W. Stephan , Science 2006, 314, 1124–1126. 17110572 10.1126/science.1134230

[5] J. J. Cabrera-Trujillo , I. Fernández , J. Phys. Chem. A 2019, 123, 10095–10101. 31689361 10.1021/acs.jpca.9b08573

[6] C. Mück-Lichtenfeld , S. Grimme , Dalton Trans. 2012, 41, 9111. 22595954 10.1039/c2dt30562c

[7] D. W. Stephan , G. Erker , Chem. Sci. 2014, 5, 2625–2641.

[8] A. J. P. Cardenas , B. J. Culotta , T. H. Warren , S. Grimme , A. Stute , R. Fröhlich , G. Kehr , G. Erker , Angew. Chem. Int. Ed. 2011, 50, 7567–7571. 10.1002/anie.201101622 21726024

[9] J. Zhu , Chem. Asian J. 2019, 14, 1304–1304.

[10] H. Mondal , S. G. Patra , P. K. Chattaraj , Struct. Chem. 2022, 33, 1853–1865.

[11] D. W. Stephan , G. Erker , Angew. Chem. Int. Ed. 2010, 49, 46–76. 10.1002/anie.200903708 20025001

[12] M. Sajid , G. Kehr , C. G. Daniliuc , G. Erker , Angew. Chem. Int. Ed. 2014, 53, 1118–1121. 10.1002/anie.201307551 24338931

[13] E. L. Kolychev , T. Bannenberg , M. Freytag , C. G. Daniliuc , P. G. Jones , M. Tamm , Chem. A Eur. J. 2012, 18, 16938–16946. 10.1002/chem.201202840 23150467

[14] C. Appelt , H. Westenberg , F. Bertini , A. W. Ehlers , J. C. Slootweg , K. Lammertsma , W. Uhl , Angew. Chem. Int. Ed. 2011, 50, 3925–3928. 10.1002/anie.201006901 21425419

[15] L. L. Liu , C. Chan , J. Zhu , C.-H. Cheng , Y. Zhao , J. Org. Chem. 2015, 80, 8790–8795. 26247714 10.1021/acs.joc.5b01726

[16] H. Mondal , S. G. Patra , P. K. Chattaraj , J. Chem. Sci. 2022, 134, 119.

[17] H. Mondal , M. Ghara , P. K. Chattaraj , Chem. Phys. Lett. 2021, 774, 138623.

[18] M. Ghara , H. Mondal , R. Pal , P. K. Chattaraj , J. Phys. Chem. A 2023, 127, 4561–4582. 37216335 10.1021/acs.jpca.3c02141

[19] S. R. Flynn , D. F. Wass , ACS Catal. 2013, 3, 2574–2581.

[20] M. Spittler , L. Helmecke , C. Czekelius , Eur. J. Org. Chem. 2019, 2019, 458–468.

[21] J. Daru , I. Bakó , A. Stirling , I. Pápai , ACS Catal. 2019, 9, 6049–6057.

[22] I. Fernández , Chem. Commun. 2022, 58, 4931–4940. 10.1039/d2cc00233g 35322823

[23] J. J. Cabrera-Trujillo , I. Fernández , Chem. A Eur. J. 2021, 27, 3823–3831. 10.1002/chem.202004733 33231334

[24] D. Yepes , P. Jaque , I. Fernández , Chem. A Eur. J. 2016, 22, 18801–18809. 10.1002/chem.201603889 27859795

[25] M. Heshmat , B. Ensing , J. Phys. Chem. A 2020, 124, 6399–6410. 32666803 10.1021/acs.jpca.0c03108 PMC8279552

[26] T. A. Rokob , A. Hamza , I. Pápai , J. Am. Chem. Soc. 2009, 131, 10701–10710. 19722636 10.1021/ja903878z

[27] B. L. Durfey , T. M. Gilbert , Inorg. Chem. 2011, 50, 7871–7879. 21774464 10.1021/ic201182p

[28] D. Wu , A. Liu , D. Jia , Comput. Theor. Chem. 2018, 1131, 33–39.

[29] A. E. Ashley , T. J. Herrington , G. G. Wildgoose , H. Zaher , A. L. Thompson , N. H. Rees , T. Krämer , D. O'Hare , J. Am. Chem. Soc. 2011, 133, 14727–14740. 21786772 10.1021/ja205037t

[30] J. A. Nicasio , S. Steinberg , B. Inés , M. Alcarazo , Chem. A Eur. J. 2013, 19, 11016–11020. 10.1002/chem.201301158 23813663

[31] Q. Yin , S. Kemper , H. F. T. Klare , M. Oestreich , Chem. A Eur. J. 2016, 22, 13840–13844. 10.1002/chem.201603466 27447683

[32] J. F. Kögel , A. Y. Timoshkin , A. Schröder , E. Lork , J. Beckmann , Chem. Sci. 2018, 9, 8178–8183. 30568768 10.1039/c8sc02981d PMC6256356

[33] C. Stoian , M. Olaru , T. A. Cucuiet , K. T. Kegyes , A. Sava , A. Y. Timoshkin , C. I. Raţ , J. Beckmann , Chem. A Eur. J. 2021, 27, 4327–4331. 10.1002/chem.202005367 PMC7986919 33368648

[34] A. Y. Timoshkin , G. Frenking , Organometallics 2008, 27, 371–380.

[35] E. I. Davydova , T. N. Sevastianova , A. Y. Timoshkin , Coord. Chem. Rev. 2015, 297–298, 91–126.

[36] L. A. Mück , A. Y. Timoshkin , G. Frenking , Inorg. Chem. 2012, 51, 640–646. 22168307 10.1021/ic202152h

[37] T. M. Gilbert , Dalton Trans. 2012, 41, 9046. 22460739 10.1039/c2dt30208j

[38] T. K. Wood , W. E. Piers , B. A. Keay , M. Parvez , Org. Lett. 2006, 8, 2875–2878. 16774279 10.1021/ol061201w

[39] B. M. Mikhailov , Pure Appl. Chem. 1980, 52, 691–704.

[40] Y. N. Bubnov , M. E. Gurskii , D. G. Pershin , K. A. Lyssenko , M. Y. Antipin , Russ. Chem. Bull. 1998, 47, 1771–1777.

[41] M. El-Hamdi , A. Y. Timoshkin , J. Comput. Chem. 2019, 40, 1892–1901. 31017697 10.1002/jcc.25845

[42] A. Chardon , A. Osi , D. Mahaut , T. Doan , N. Tumanov , J. Wouters , L. Fusaro , B. Champagne , G. Berionni , Angew. Chem. Int. Ed. 2020, 59, 12402–12406. 10.1002/anie.202003119 32324961

[43] A. Ben Saida , A. Chardon , A. Osi , N. Tumanov , J. Wouters , A. I. Adjieufack , B. Champagne , G. Berionni , Angew. Chem. Int. Ed. 2019, 58, 16889–16893. 10.1002/anie.201910908 31475396

[44] D. W. Stephan , G. Erker , Angew. Chem. Int. Ed. 2015, 54, 6400–6441. 10.1002/anie.201409800 25974714

[45] A. Hamza , K. Sorochkina , B. Kótai , K. Chernichenko , D. Berta , M. Bolte , M. Nieger , T. Repo , I. Pápai , ACS Catal. 2020, 10, 14290–14301.

[46] J. Lam , K. M. Szkop , E. Mosaferi , D. W. Stephan , Chem. Soc. Rev. 2019, 48, 3592–3612. 30178796 10.1039/c8cs00277k

[47] S. Tussing , L. Greb , S. Tamke , B. Schirmer , C. Muhle-Goll , B. Luy , J. Paradies , Chem. A Eur. J. 2015, 21, 8056–8059. 10.1002/chem.201500805 25877865

[48] L. Greb , S. Tussing , B. Schirmer , P. Oña-Burgos , K. Kaupmees , M. Lõkov , I. Leito , S. Grimme , J. Paradies , Chem. Sci. 2013, 4, 2788.

[49] S. Tussing , K. Kaupmees , J. Paradies , Chem. A Eur. J. 2016, 22, 7422–7426. 10.1002/chem.201600716 27060884

[50] D. J. Scott , N. A. Phillips , J. S. Sapsford , A. C. Deacy , M. J. Fuchter , A. E. Ashley , Angew. Chem. 2016, 128, 14958–14962. 10.1002/anie.201606639 PMC5129554 27774711

[51] M.-A. Courtemanche , A. P. Pulis , É. Rochette , M.-A. Légaré , D. W. Stephan , F.-G. Fontaine , Chem. Commun. 2015, 51, 9797–9800. 10.1039/c5cc03072b 25994329

[52] M. J. Frisch, G. W. Trucks, H. B. Schlegel, G. E. Scuseria, M. A. Robb, J. R. Cheeseman, G. Scalmani, V. Barone, G. A. Petersson, H. Nakatsuji, X. Li, M. Caricato, A. V Marenich, J. Bloino, B. G. Janesko, R. Gomperts, B. Mennucci, H. P. Hratchian, J. V Ortiz, A. F. Izmaylov, J. L. Sonnenberg, D. Williams-Young, F. Ding, F. Lipparini, F. Egidi, J. Goings, B. Peng, A. Petrone, T. Henderson, D. Ranasinghe, V. G. Zakrzewski, J. Gao, N. Rega, G. Zheng, W. Liang, M. Hada, M. Ehara, K. Toyota, R. Fukuda, J. Hasegawa, M. Ishida, T. Nakajima, Y. Honda, O. Kitao, H. Nakai, T. Vreven, K. Throssell, J. A. Montgomery Jr., J. E. Peralta, F. Ogliaro, M. J. Bearpark, J. J. Heyd, E. N. Brothers, K. N. Kudin, V. N. Staroverov, T. A. Keith, R. Kobayashi, J. Normand, K. Raghavachari, A. P. Rendell, J. C. Burant, S. S. Iyengar, J. Tomasi, M. Cossi, J. M. Millam, M. Klene, C. Adamo, R. Cammi, J. W. Ochterski, R. L. Martin, K. Morokuma, O. Farkas, J. B. Foresman, D. J. Fox, Gaussian 16, Rev. C.01, Gaussian, Inc., Wallingford CT **2016**.

[53] M. Walker , A. J. A. Harvey , A. Sen , C. E. H. Dessent , J. Phys. Chem. A 2013, 117, 12590–12600. 24147965 10.1021/jp408166m

[54] B. Mennucci , WIREs Comput. Mol. Sci. 2012, 2, 386–404.

[55] A. Hellweg , D. Rappoport , Phys. Chem. Chem. Phys. 2015, 17, 1010–1017. 25410795 10.1039/c4cp04286g

[56] E. D. Glendening , C. R. Landis , F. Weinhold , J. Comput. Chem. 2013, 34, 1429–1437. 23483590 10.1002/jcc.23266

[57] C. Y. Legault, CYLview, 1.0b. Université de Sherbrooke, 2009. http://www.cylview.org>.

[58] D. H. Ess , K. N. Houk , J. Am. Chem. Soc. 2007, 129, 10646–10647. 17685614 10.1021/ja0734086

[59] F. M. Bickelhaupt , J. Comput. Chem. 1999, 20, 114–128.

[60] I. Fernández , F. M. Bickelhaupt , Chem. Soc. Rev. 2014, 43, 4953–4967. 24699791 10.1039/c4cs00055b

[61] W.-J. van Zeist , F. M. Bickelhaupt , Org. Biomol. Chem. 2010, 8, 3118. 20490400 10.1039/b926828f

[62] M. von Hopffgarten , G. Frenking , WIREs Comput. Mol. Sci. 2012, 2, 43–62.

[63] F. M. Bickelhaupt , E. J. Baerends , Kohn-Sham Density Functional Theory: Predicting and Understanding Chemistry, 2000.

[64] G. Frenking , F. Matthias Bickelhaupt , The EDA Perspective of Chemical Bonding, in The Chemical Bond: Fundamental Aspects of Chemical Bonding, Wiley-VCH: Weinheim, 2014, 121.

[65] L. Zhao , M. von Hopffgarten , D. M. Andrada , G. Frenking , WIREs Comput. Mol. Sci. 2018, 8, DOI 10.1002/wcms.1345.

[66] L. Zhao , S. Pan , N. Holzmann , P. Schwerdtfeger , G. Frenking , Chem. Rev. 2019, 119, 8781–8845. 31251603 10.1021/acs.chemrev.8b00722

[67] F. M. Bickelhaupt , C. Fonseca Guerra , M. Mitoraj , F. Sagan , A. Michalak , S. Pan , G. Frenking , Phys. Chem. Chem. Phys. 2022, 24, 15726–15735. 35730200 10.1039/d2cp02153f

[68] L. Zhao , S. Pan , G. Frenking , Energy Decomposition Analysis of the Chemical Bond: Scope and Limitation, in Reference Module in Chemistry, Molecular Sciences and Chemical Engineering, Elsevier, 2022.

[69] J. Tirado-Rives , W. L. Jorgensen , J. Chem. Theory Comput. 2008, 4, 297–306. 26620661 10.1021/ct700248k

[70] S. Grimme , J. Antony , S. Ehrlich , H. Krieg , J. Chem. Phys. 2010, 132, DOI 10.1063/1.3382344. 20423165

[71] Pauling L , The Nature of the Chemical Bond, Cornell University Press, Ithica, NY, 1960.

[72] R. G. Pearson , Science 1966, 151, 172–177. 17746330 10.1126/science.151.3707.172

[73] R. G. Parr , R. A. Donnelly , M. Levy , W. E. Palke , J. Chem. Phys. 1978, 68, 3801–3807.

[74] R. G. Parr , R. G. Pearson , J. Am. Chem. Soc. 1983, 105, 7512–7516.

[75] R. Das , J.-L. Vigneresse , P. K. Chattaraj , Int. J. Quantum Chem. 2014, 114, 1421–1429.

[76] R. G. Parr , L. v. Szentpály , S. Liu , J. Am. Chem. Soc. 1999, 121, 1922–1924.

[77] P. K. Chattaraj , D. R. Roy , Chem. Rev. 2007, 107, PR46–PR74.

[78] D. Chakraborty , P. K. Chattaraj , Chem. Sci. 2021, 12, 6264–6279. 34084424 10.1039/d0sc07017c PMC8115084

[79] P. K. Chattaraj , S. Giri , S. Duley , Chem. Rev. 2011, 111, PR43–PR75. 21306180 10.1021/cr100149p

[80] K. Fukui , Science 1982, 218, 747–754. 17771019 10.1126/science.218.4574.747

[81] W. Yang , W. J. Mortier , J. Am. Chem. Soc. 1986, 108, 5708–5711. 22175316 10.1021/ja00279a008

[82] P. Geerlings , E. Chamorro , P. K. Chattaraj , F. De Proft , J. L. Gázquez , S. Liu , C. Morell , A. Toro-Labbé , A. Vela , P. Ayers , Theor. Chem. Acc. 2020, 139, 36.

[83] Conceptual Density Functional Theory: Towards a New Chemical Reactivity TheoryTitle (Ed.: S. Liu ), Wiley-VCH, 2022.

[84] P. K. Chattaraj , B. Maiti , U. Sarkar , J. Phys. Chem. A 2003, 107, 4973–4975.

[85] D. R. Roy , R. Parthasarathi , J. Padmanabhan , U. Sarkar , V. Subramanian , P. K. Chattaraj , J. Phys. Chem. A 2006, 110, 1084–1093. 16420012 10.1021/jp053641v

[86] B. Cordero , V. Gómez , A. E. Platero-Prats , M. Revés , J. Echeverría , E. Cremades , F. Barragán , S. Alvarez , Dalton Trans. 2008, 2832. 18478144 10.1039/b801115j

[87] J.-X. Zhang , F. K. Sheong , Z. Lin , Chem. A Eur. J. 2018, 24, 9639–9650. 10.1002/chem.201801220 29667258

[88] J. Zhang , F. K. Sheong , Z. Lin , WIREs Comput. Mol. Sci. 2020, 10, DOI 10.1002/wcms.1469.

[89] A. Hamza , A. Stirling , T. András Rokob , I. Pápai , Int. J. Quantum Chem. 2009, 109, 2416–2425.

[90] J. Sánchez-Márquez , H. Mondal , S. G. Patra , A. Morales-Bayuelo , P. K. Chattaraj , Int. J. Quantum Chem. 2023, 123, DOI 10.1002/qua.27129.

[91] S. G. Patra , R. Jha , H. Mondal , P. K. Chattaraj , J. Phys. Org. Chem. 2022, DOI 10.1002/poc.4337.

[92] S. G. Patra , H. Mondal , P. K. Chattaraj , J. Phys. Org. Chem. 2022, DOI 10.1002/poc.4359.

