# Supplementary material for: Unraveling Reactivity Pathways: Dihydrogen Activation and Hydrogenation of Multiple Bonds by Pyramidalized Boron‐Based Frustrated Lewis Pairs
Source: ChemistryOpen. 2023 Dec 20;13(4):e202300179. doi: 10.1002/open.202300179 (PMC11004477; doi:10.1002/open.202300179)
Supplement: Supplementary file 1 — Supporting Information [file OPEN-13-e202300179-s001.pdf]

ChemistryOpen

Supporting Information

Unraveling Reactivity Pathways: Dihydrogen Activation and Hydrogenation of Multiple Bonds by Pyramidalized Boron-Based Frustrated Lewis Pairs

Himangshu Mondal and Pratim Kumar Chattaraj*

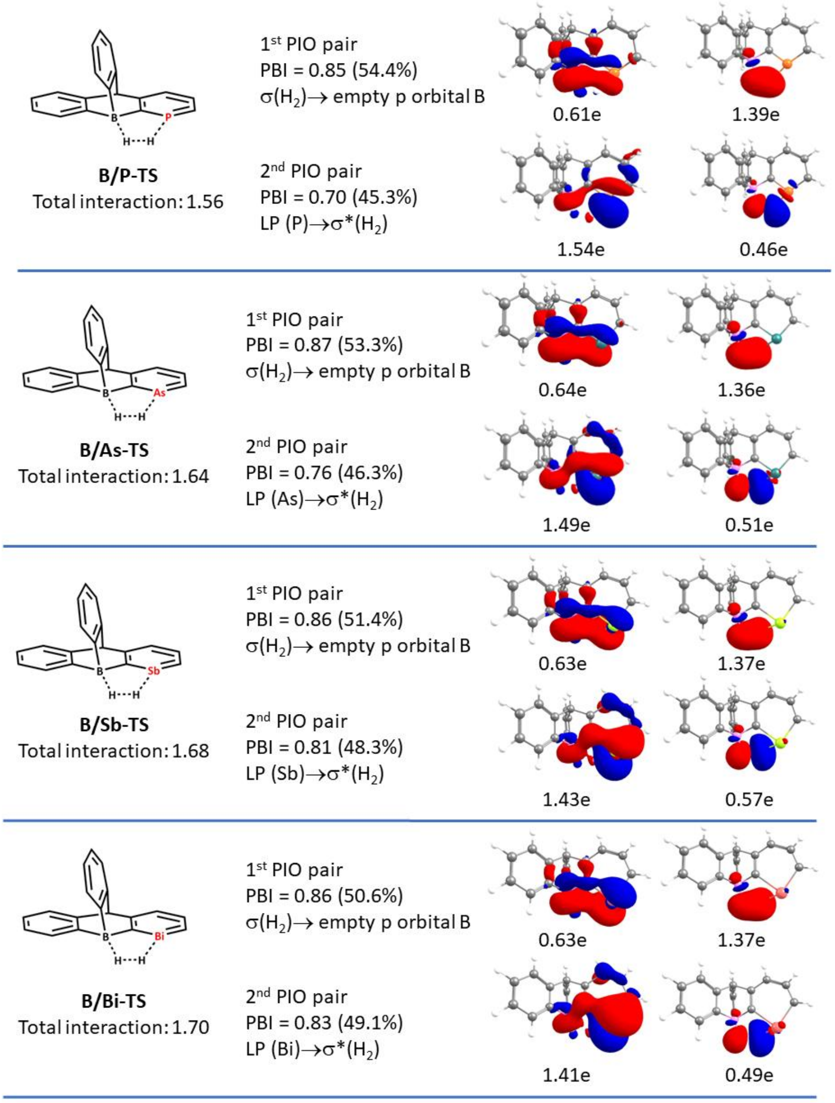

Figure S1. *PIO analysis of B/E-TSs using B/E-FLP and H_2 as two fragments. PBI and its percentage contribution to the overall interactions between two are also shown. The isovalue is 0.03.*

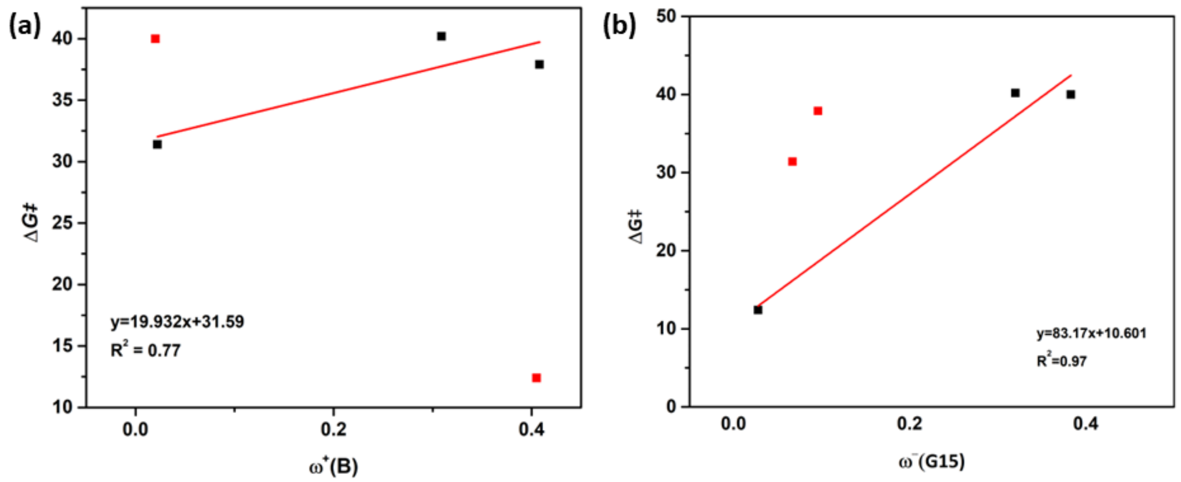

Figure S2. *Comparison of Activation Energy with (a) Local Electrophilicity at Boron Centers (ω+) and (b) Local Nucleophilicity at Group 15 Centers (ω-). Data points in red colors are not included in the linear fit.*

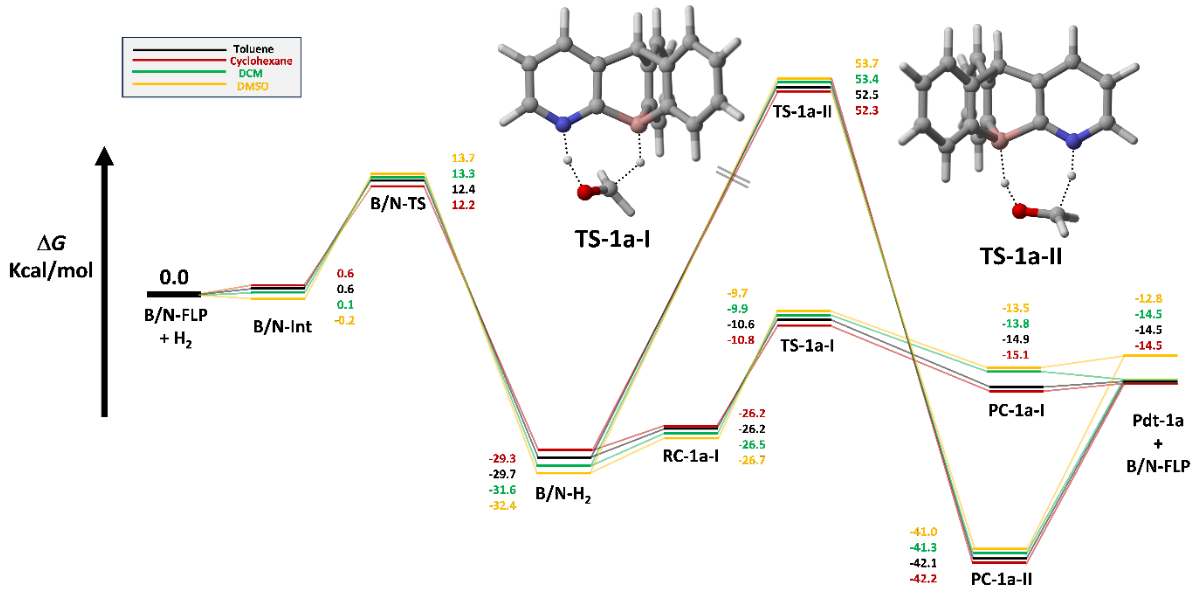

Figure S3. *Computed solvent corrected relative free energy profile for the hydrogenation reaction of formaldehyde (**1a**) by B/N-FLP. All data have been computed at the PCM-(solvent)-M06-2X/def2-TZVP// M06-2X/def2-SVP level of theory (toluene = black, cyclohexane = brown, dichloromethane (DCM) = green and dimethyl-sulfoxide (DMSO) = orange)*

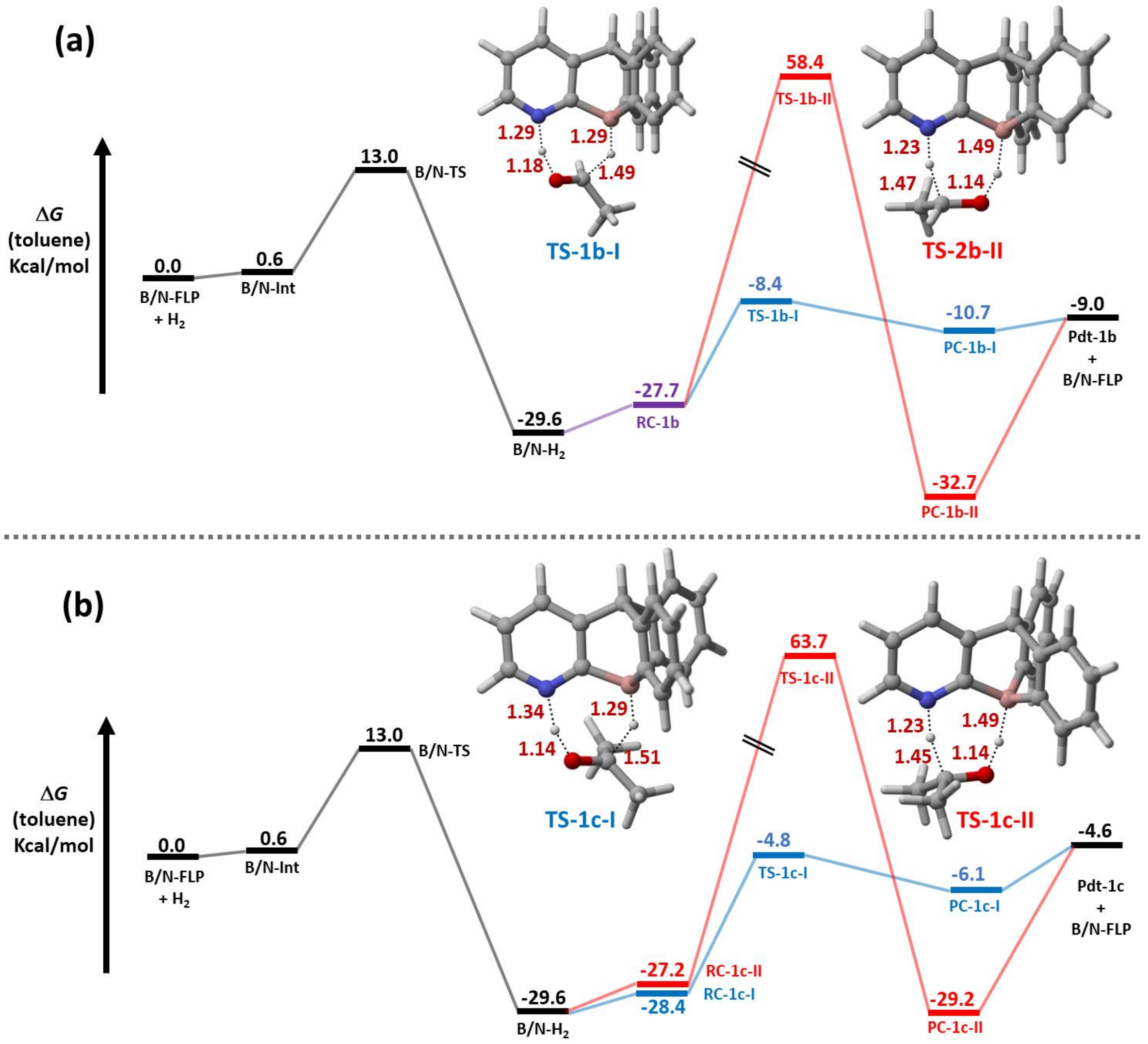

Figure S4. *Computed solvent corrected relative free energy profile for the hydrogenation reaction of (a) acetaldehyde (**1b**) (b) ketone (**1c**) by B/N-FLP. Relative free energies (ΔG, computed at 298 K) and bond lengths are given in kcal/mol and Å, respectively. All data have been computed at the PCM-(toluene)-M06-2X/def2-TZVP// M06-2X/def2-SVP level of theory*

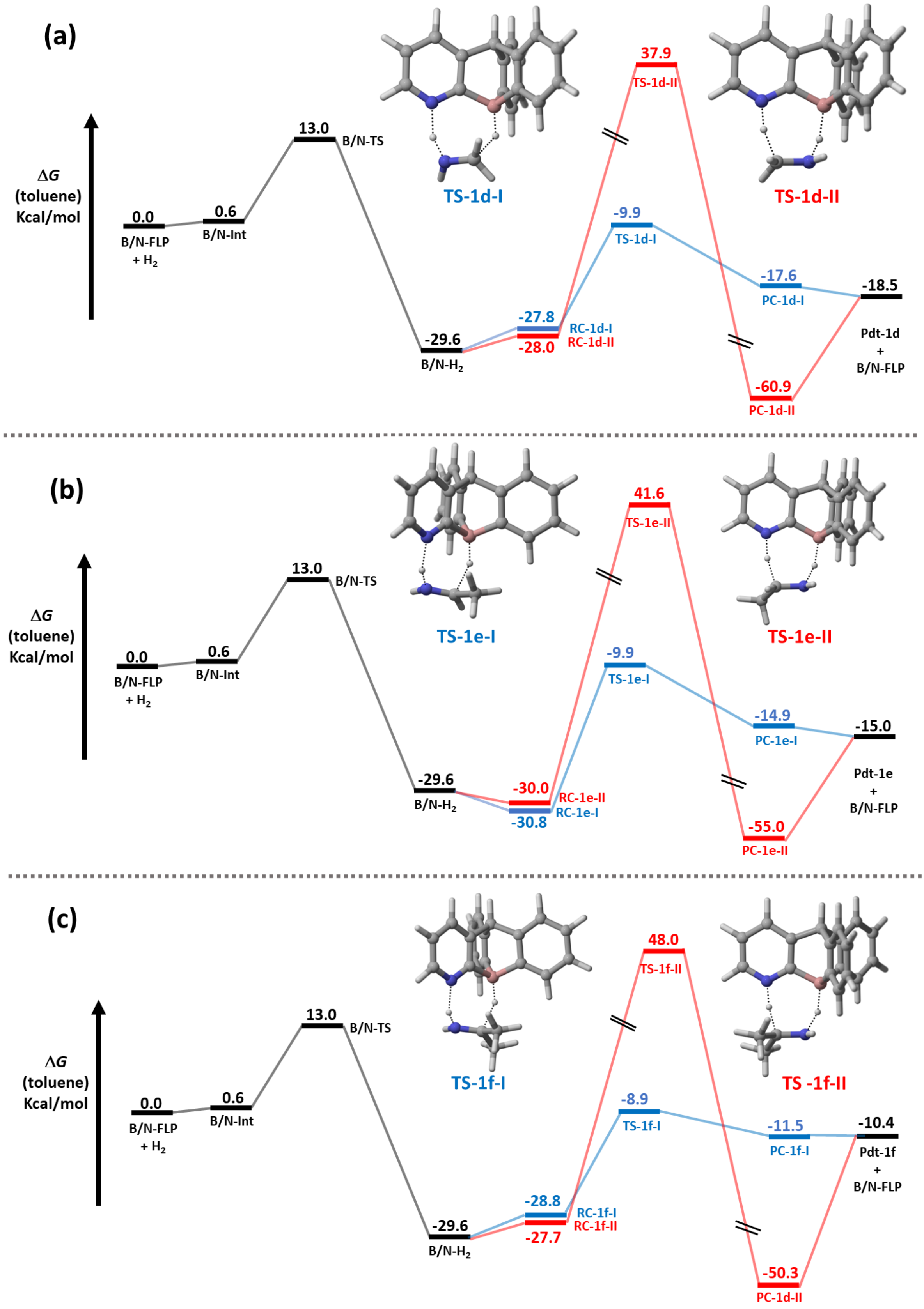

(a)
ΔG
(toluene)
Kcal/mol
0.0
B/N-FLP
+ H2
0.6
B/N-Int
13.0
B/N-TS
-29.6
B/N-H2
-27.8
RC-1d-I
-28.0
RC-1d-II
37.9
TS-1d-II
-9.9
TS-1d-I
-17.6
PC-1d-I
-18.5
Pdt-1d
+
B/N-FLP
-60.9
PC-1d-II
TS-1d-I
TS-1d-II
(b)
ΔG
(toluene)
Kcal/mol
0.0
B/N-FLP
+ H2
0.6
B/N-Int
13.0
B/N-TS
-29.6
B/N-H2
-30.0
RC-1e-II
-30.8
RC-1e-I
41.6
TS-1e-II
-9.9
TS-1e-I
-14.9
PC-1e-I
-15.0
Pdt-1e
+
B/N-FLP
-55.0
PC-1e-II
TS-1e-I
TS-1e-II
(c)
ΔG
(toluene)
Kcal/mol
0.0
B/N-FLP
+ H2
0.6
B/N-Int
13.0
B/N-TS
-29.6
B/N-H2
-28.8
RC-1f-I
-27.7
RC-1f-II
48.0
TS-1f-II
-8.9
TS-1f-I
-11.5
PC-1f-I
-10.4
Pdt-1f
+
B/N-FLP
-50.3
PC-1d-II
TS-1f-I
TS -1f-II

Figure S5. *Computed solvent corrected relative free energy profile for the hydrogenation reaction of (a)* ***1d*** *(b)* ***1e*** *(c)* ***1f*** *by B/N-FLP. Relative free energies (ΔG, computed at 298 K). All data have been computed at the PCM-(toluene)-M06-2X/def2-TZVP// M06-2X/def2-SVP level of theory*

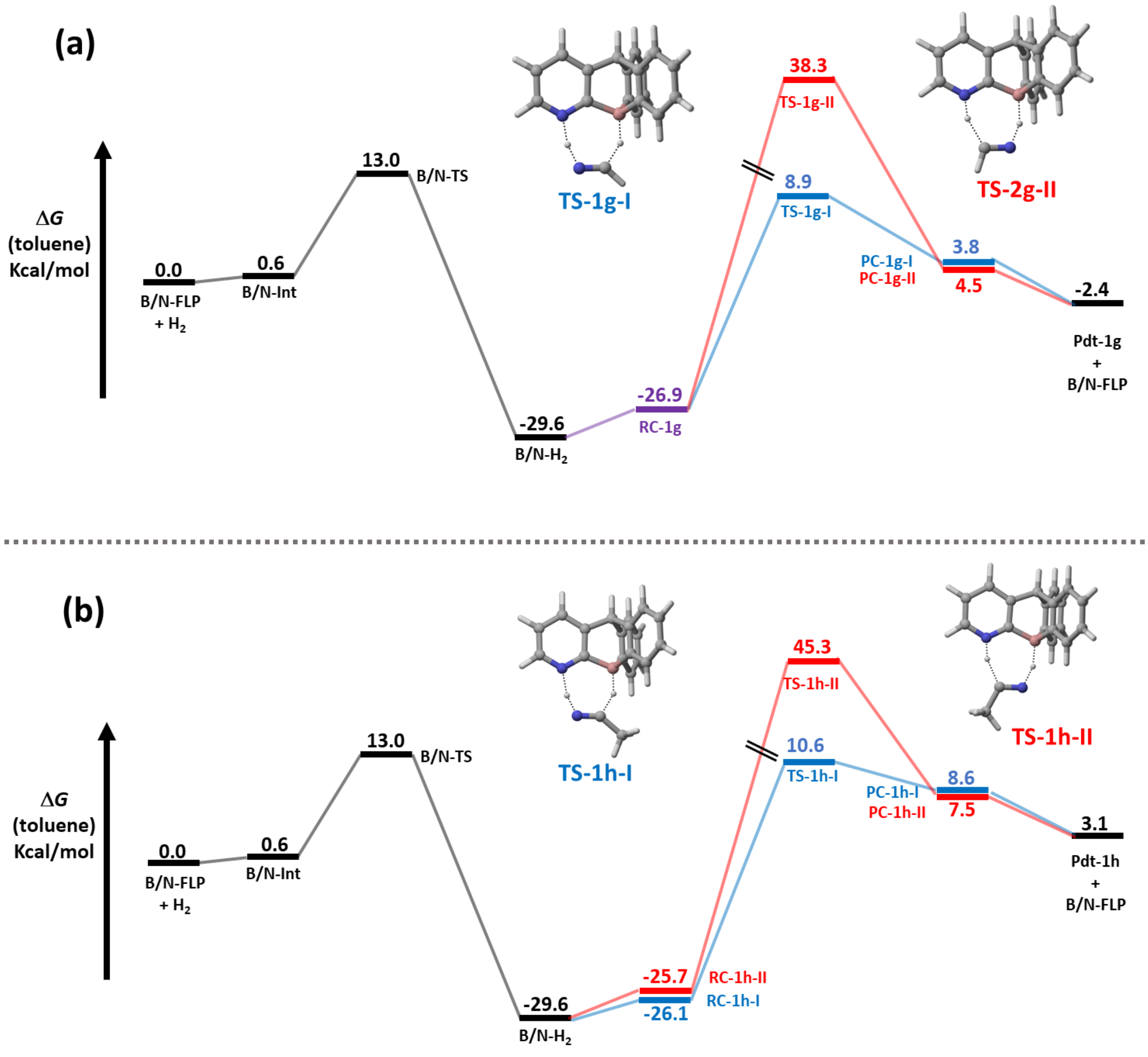

Figure S6. *Computed solvent corrected relative free energy profile for the hydrogenation reaction of (a)* ***1g*** *(b)* ***1h*** *by B/N-FLP. Relative free energies (ΔG, computed at 298 K). All data have been computed at the PCM-(toluene)-M06-2X/def2-TZVP// M06-2X/def2-SVP level of theory*

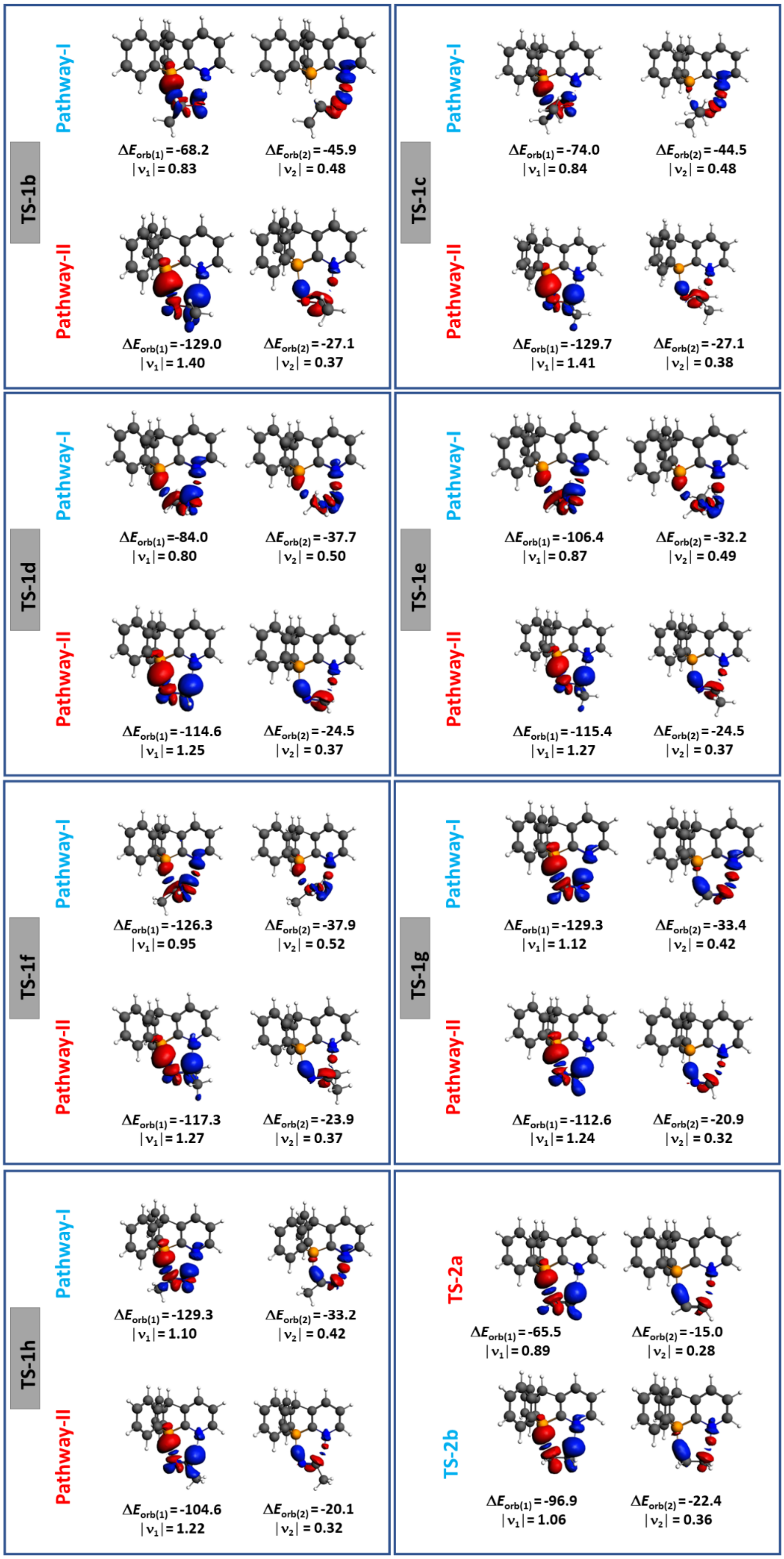

TS-1b
Pathway-I
ΔE_orb(1) = -68.2
|ν1| = 0.83
ΔE_orb(2) = -45.9
|ν2| = 0.48
Pathway-II
ΔE_orb(1) = -129.0
|ν1| = 1.40
ΔE_orb(2) = -27.1
|ν2| = 0.37
TS-1c
Pathway-I
ΔE_orb(1) = -74.0
|ν1| = 0.84
ΔE_orb(2) = -44.5
|ν2| = 0.48
Pathway-II
ΔE_orb(1) = -129.7
|ν1| = 1.41
ΔE_orb(2) = -27.1
|ν2| = 0.38
TS-1d
Pathway-I
ΔE_orb(1) = -84.0
|ν1| = 0.80
ΔE_orb(2) = -37.7
|ν2| = 0.50
Pathway-II
ΔE_orb(1) = -114.6
|ν1| = 1.25
ΔE_orb(2) = -24.5
|ν2| = 0.37
TS-1e
Pathway-I
ΔE_orb(1) = -106.4
|ν1| = 0.87
ΔE_orb(2) = -32.2
|ν2| = 0.49
Pathway-II
ΔE_orb(1) = -115.4
|ν1| = 1.27
ΔE_orb(2) = -24.5
|ν2| = 0.37
TS-1f
Pathway-I
ΔE_orb(1) = -126.3
|ν1| = 0.95
ΔE_orb(2) = -37.9
|ν2| = 0.52
Pathway-II
ΔE_orb(1) = -117.3
|ν1| = 1.27
ΔE_orb(2) = -23.9
|ν2| = 0.37
TS-1g
Pathway-I
ΔE_orb(1) = -129.3
|ν1| = 1.12
ΔE_orb(2) = -33.4
|ν2| = 0.42
Pathway-II
ΔE_orb(1) = -112.6
|ν1| = 1.24
ΔE_orb(2) = -20.9
|ν2| = 0.32
TS-1h
Pathway-I
ΔE_orb(1) = -129.3
|ν1| = 1.10
ΔE_orb(2) = -33.2
|ν2| = 0.42
Pathway-II
ΔE_orb(1) = -104.6
|ν1| = 1.22
ΔE_orb(2) = -20.1
|ν2| = 0.32
TS-2a
ΔE_orb(1) = -65.5
|ν1| = 0.89
ΔE_orb(2) = -15.0
|ν2| = 0.28
TS-2b
ΔE_orb(1) = -96.9
|ν1| = 1.06
ΔE_orb(2) = -22.4
|ν2| = 0.36

Figure S7. *The shape of the deformation density* $\Delta\rho_{(1)}$ *and* $\Delta\rho_{(2)}$*, is associated with the orbital interaction* $\Delta E_{orb(1)}$ *and* $\Delta E_{orb(2)}$ *in the TSs and eigenvalues* $|\nu_n|$ *of the charge flow. The isosurface value is 0.003. The color code of the charge flow is red→blue.*
